# Musculoskeletal magnetic resonance imaging in the DE50-MD dog model of Duchenne muscular dystrophy

**DOI:** 10.1016/j.nmd.2021.05.010

**Published:** 2021-08

**Authors:** Natasha L. Hornby, Randi Drees, Rachel Harron, Ruby Chang, Dominic J. Wells, Richard J. Piercy

**Affiliations:** a Comparative Neuromuscular Diseases Laboratory, Department of Clinical Sciences and Services, Royal Veterinary College, London NW1 0TU, United Kingdom; b Queen Mother Hospital for Animals, Department of Clinical Sciences and Services, Royal Veterinary College, Hatfield AL9 7TA, United Kingdom; c Comparative Biomedical Sciences, Royal Veterinary College, London NW1 0TU, United Kingdom

**Keywords:** DMD, DE50-MD, Imaging biomarkers, MRI, Musculoskeletal

## Abstract

The DE50-MD canine model of Duchenne muscular dystrophy (DMD) has a dystrophin gene splice site mutation causing deletion of exon 50, an out-of-frame transcript and absence of dystrophin expression in striated muscles. We hypothesized that the musculoskeletal phenotype of DE50-MD dogs could be detected using Magnetic Resonance Imaging (MRI), that it would progress with age and that it would reflect those in other canine models and DMD patients. 15 DE50-MD and 10 age-matched littermate wild type (WT) male dogs underwent MRI every 3 months from 3 to 18 months of age. Normalized muscle volumes, global muscle T2 and ratio of post- to pre-gadolinium T1-weighted SI were evaluated in 7 pelvic limb and 4 lumbar muscles bilaterally. DE50-MD dogs, compared to WT, had smaller volumes in all muscles, except the cranial sartorius; global muscle T2 was significantly higher in DE50-MD dogs compared to WT. Muscle volumes plateaued and global muscle T2 decreased with age. Normalized muscle volumes and global muscle T2 revealed significant differences between groups longitudinally and should be useful to determine efficacy of therapeutics in this model with suitable power and low sample sizes. Musculoskeletal changes reflect those of DMD patients and other dog models.

## Highlights

• Normalized muscle volumes distinguish between wild type and DE50-MD dogs.
• Global muscle T2 signal intensities discriminate between wild type and DE50-MD dogs.
• Musculoskeletal changes detected by MRI reflect those seen in human DMD patients.
• Musculoskeletal MRI in this model will be useful to assess treatment efficacy.

## 1 Introduction

Duchenne muscular dystrophy (DMD), an X-linked, recessive, fatal, muscle-wasting disease that affects approximately 1 in 5000 boys is caused by null mutations in the gene that encodes for dystrophin [1], [2], [3]. Absence of dystrophin protein in skeletal muscles results in muscle fibre membrane instability during normal muscle contraction and cell signalling defects; leading to necrosis, inflammation, cycles of degeneration and regeneration, muscle atrophy, accumulation of endomysial collagen and fat and progressive paresis [4], [5], [6]. Despite promising developments with gene therapies, many DMD clinical trials continue to use animal models prior to translation to human patients.

There are several animal models used in DMD research, including the *mdx* mouse and the Golden Retriever Muscular Dystrophy (GRMD) dog. The GRMD dog model (with a splice site mutation in intron 6) has historically been the most widely used canine model of DMD [7], [8], [9], [10]. Beagle crosses (with the same mutation) have been created to develop a separate, smaller line of dogs in Japan, known as CXMD_J_ [11]. When compared to *mdx* mice that have minimal clinically applicable musculoskeletal features, canine models more closely reflect the human phenotype, both functionally and histologically [7], [8], [9],[12], [13], [14]. In 2010, we reported a spontaneous (splice site) mutation in a Cavalier King Charles Spaniel that results in deletion of exon 50 and an out of frame transcript [15]. This mutation, which is at the center of the region of the dystrophin gene that is most commonly mutated in DMD patients [3,[16], [17], [18], has since been maintained on a beagle background to create a unique colony, known as the DE50-MD dog. Recently, in collaboration, we reported the first successful use of systemic CRISPR/Cas9 mediated gene editing for DMD in a large animal, in dogs from this colony [19].

Skeletal muscle histopathology is an important tool for monitoring disease progression in boys with DMD. However, muscle biopsy is invasive and can only provide limited local information that might not be representative of the muscle in its entirety. In contrast, magnetic resonance imaging (MRI) is a valuable non-invasive technique for monitoring the global progression of disease in the skeletal muscle of human DMD patients [20]. In GRMD dog models, MRI has been used to monitor disease progression and compare affected dogs with wild type (WT) littermate dogs [9,[21], [22], [23], [24], [25], [26], [27], [28], with thoracic and pelvic limb muscles assessed.

Most notably, individual muscle total volumes have been obtained from standard T1-weighted (T1w) sequences, revealing smaller thoracic and pelvic limb muscle volumes in affected GRMD dogs when compared to WT dogs, apart from in the cranial sartorius muscle [21], [22], [23], [24], [25], [26]. A few previous canine model studies have utilized semi-automated muscle segmentation to expedite data collection of muscle volumes with accurate results [25,26]. Global, water and fat T2 map signal intensities (SI) are valuable quantitative measurements in DMD boys [29], [30], [31], [32], [33] and similarly, in GRMD dogs [22,^23^,^25^,26]. Most recently, Dixon sequences and water T2 maps are the MRI sequences of choice in DMD patients for longitudinal monitoring [20,[34], [35], [36], [37], [38], [39], [40].

A ‘heterogeneity index’ used to measure T2-weighted (T2w) heterogeneity has been used in the GRMD dog model where affected dogs have higher values than WT animals, probably due to increased intramuscular fibrosis and less likely fat [26]. Increased T2w SI and decreased T1w SI in affected GRMD dog muscle compared to WT dogs, most likely resulted from “oedema-like lesions” rather than fat infiltration, as fat would also generate T1 hyperintensities, which were not observed [24]. In that same study, there were no significant differences for fat saturated images compared to non-fat saturated images in either group [24], reflecting the fact that fat replacement is much less prominent in these relatively young canine dogs. Affected GRMD dog skeletal muscles also have a higher SI in post-gadolinium T1w images than WT dogs [21–27]. Additionally, in DMD patients, non-fat saturated T2 map images correlated better to functional measures than images obtained from fat saturated T2 map sequences [32].

The main objective of this study was to establish if the musculoskeletal MRI phenotype of DE50-MD dogs could be detected with MRI and used to aid monitoring of disease severity, extent and progression in the DE50-MD dog model. Additionally, we hypothesised that the disease would progress with age and features would reflect those in other canine models and boys with DMD.

## 2 Materials and methods

### 2.1 Animals and anesthesia

This study was conducted within a UK Animals (Scientific Procedures) Act 1986 (ASPA) project license and with approval by the local Animal Welfare Ethical Review Board (AWERB). Adult dogs were group housed (12-hour light/dark cycle; 15–24 °C) in large kennels, until pregnant females were close to whelping when they were housed singly. Puppies were kept with their mother up to weaning at 10–12 weeks old, then grouped in their litters until approximately 4/5 months of age. Adults were then maintained in groups of 3 or 4 animals. Dogs were fed Burns puppy or adult feed twice a day *ad lib*, with daily human interaction and access to outdoor runs and grassy paddocks: conditions that exceed the minimum Animals (Scientific Procedures) Act 1986 (ASPA) requirements. Carrier female dogs were naturally mated with WT beagle males (RCC strain). Male puppies born into the colony between 2015 and 2018 were included in the study in date of birth order. Puppy genotypes were confirmed by sequencing of PCR products amplified from cheek swab-derived DNA within 7 days from birth and corroborated by measurement of serum creatine kinase activity [41]. Unaffected animals, not used for research, were rehomed.

Imaging investigations were performed in a total of 15 DE50-MD dogs and 10 age-matched littermate WT male dogs every 3 months, from 3 to 18 months of age under general anesthesia, though not all dogs were included at every time point. Dogs were premedicated with 0.2 mg/kg methadone (Synthadon, Animalcare), induced with 4–6 mg/kg propofol to effect (Propoflo, Zoetis), intubated and maintained on an inhalational mixture of sevoflurane (SevoFlo, Zoetis) and oxygen whilst being infused with 5 ml/kg/hr Hartmann's solution (Aquapharm11, Animalcare).

### 2.2 MRI acquisition

Dogs were scanned in dorsal recumbency using a 1.5T Philips Intera MRI scanner (Philips Medical Systems, Best, The Netherlands). The pelvic limbs were extended, and the metatarsi were positioned over the level of element 1 of the spine coil and the tibia and femorotibial joints (stifles) were positioned over element 2 of the spine coil. Sagittal, dorsal and transverse survey images were acquired, including at least the thirteenth thoracic vertebrae (T13) cranially and stifles distally. Sagittal T1w and T2w images were acquired of the lumbar spine from T13 to the coxofemoral joints. Standard transverse T1w and T2w turbo spin echo (TSE) images were acquired of the entire lumbar spine and the entire proximal pelvic limbs separately, specifically, from cranial to T13 to the ischium caudally for the lumbar spine and including the iliac crest and stifles for the proximal pelvic limbs. Dorsal T2w thin slice gradient echo (BALTGRAD) sequences were acquired to include bilateral femurs. Global muscle T2 maps of the proximal pelvic limbs were acquired by obtaining a multi spin echo (MSE) T2w sequence (4 echoes); slices were acquired in the transverse plane at the level of mid-femur to include the entire shaft of the femur and all musculature. Post-gadolinium transverse T1w TSE sequence acquisitions of the lumbar spine and proximal pelvic limbs were acquired starting 5 min after rapid intravenous injection of 0.1 ml/kg gadolinium contrast agent **(**Gadovist, Bayer). Imaging parameters are listed in Table 1.

**Table 1:** 1.5T Philips Intera MRI sequence acquisition parameters. TE: echo time; TR: repetition time; FA: flip angle; Sag: Sagittal; Tra: Transverse; TSE: Turbo spin echo; MSE: Multi spin echo; GRE: Gradient echo; gad: gadolinium.

Sequence	Area scanned	TE (ms)	TR (ms)	Slice thickness (mm)	No. of slices	FA (°)	Field of view (mm^2^)	Matrix	In Plane Resolution (mm^2^)	Approx. scan time (min)
**Sag. T1w** **TSE**	Lumbar & Pelvic limb	8	400	2	17	90	175 × 300	196 × 336	0.89 × 0.89	2.5
**Sag. T2w** **TSE**	Lumbar & Pelvic limb	120	3000	2	17	90	175 × 300	196 × 336	0.89 × 0.89	4.5
**Tra. T1w** **TSE**	Lumbar Pelvic limb	8 8	400–600 400–600	3 3	65 45	90 90	161 × 161 155 × 175	232 × 176 220 × 192	0.69 × 0.91 0.70 × 0.91	5.0
**Tra. T2w** **TSE**	Lumbar Pelvic limb	120 120	3000–4000 3000–4000	3 3	65 45	90 90	161 × 161 155 × 175	232 × 176 220 × 192	0.69 × 0.91 0.70 × 0.91	5.0
**MSE T2 map**	Pelvic limb	20–80	1500–2000	4	18	90	159 × 159	204 × 178	0.78 × 0.89	5.5
**BAL TGRAD**	Pelvic limb	4	8	1	120	60	145 × 155	196 × 336	0.74 × 0.46	6.0
**Tra T1w +** **gad TSE**	Lumbar Pelvic limb	8 8	400–600 400–600	3 3	65 45	90 90	161 × 161 155 × 175	232 × 176 220 × 192	0.69 × 0.91 0.70 × 0.91	5.0

### 2.3 MRI image analysis

**Fig 1:**
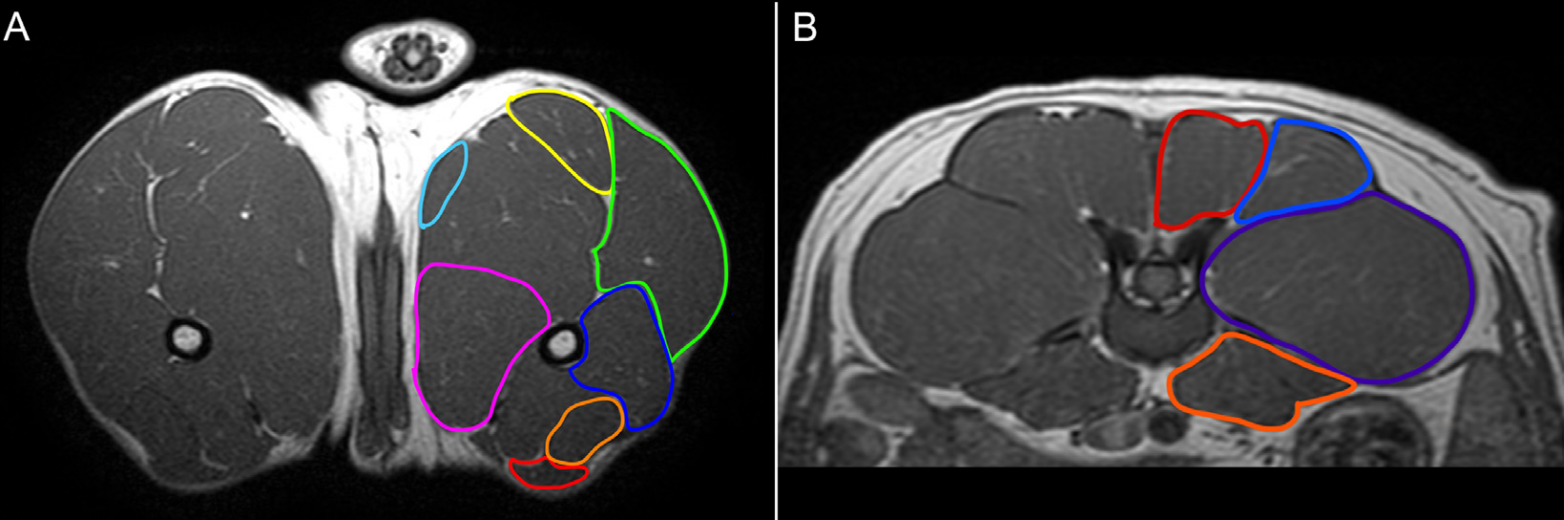
(A) Pelvic limb muscles of 12-month-old wildtype (WT) dog on T1-weighted sequence: cranial sartorius (red), rectus femoris (orange), vastus lateralis (dark blue), biceps femoris (green), semitendinosus (yellow), gracilis (light blue), adductor (pink); (B) Lumbar muscles of 12-month-old wildtype dog on T1-weighted sequence: longissimus lumborum (red), multifidus lumborum (mid-blue), iliocostalis lumborum (purple), iliopsoas (dark orange).

In both groups for each dog, at all available time-points, up to 7 pelvic limb muscles and 4 lumbar muscles were analyzed bilaterally for all musculoskeletal MRI biomarkers. The pelvic limb muscles analyzed included the cranial sartorius (CS), rectus femoris (RF), biceps femoris (BF), semitendinosus (ST), gracilis (G), adductor magnus (AD) and vastus lateralis (VL) muscles [Fig. 1.]. Additionally, muscles of the lumbar spine were examined, including the longissimus lumborum (LL), multifidus lumborum (ML), iliocostalis lumborum (IC) and iliopsoas (PS) muscles [Fig. 1.]. All analyses were performed using OsiriX/Horos DICOM viewing software (Free open source code software, horosproject.org).

#### 2.3.1 Muscle regions of interest (ROIs)

Semi-automated full-length segmentation of 6 proximal pelvic limb muscles (CS, RF, BF, ST, G and AD) and 4 lumbar muscles (LL, ML, IC and PS) bilaterally was performed: outlines of each muscle in every fifth slice were manually delineated and ROIs were generated for the intervening slices, these were manually altered to the correct muscle area on each slice as needed.

#### 2.3.2 Mean absolute muscle volume

A volume interpolation method was used to determine each muscle's volume from the ROIs. The entire muscle volume was recorded for each pelvic limb muscle bilaterally and a mean of the two calculated. Lumbar muscle volumes were measured from the cranial epiphysis of the second lumbar vertebra (L2) to the caudal epiphysis of the fifth lumbar vertebra (L5) bilaterally, and the mean calculated.

#### 2.3.3 Mean muscle signal intensity (SI)

The full-length muscle volume ROIs generated in the CS, RF, BF, ST, G, AD, LL, ML, IC and PS muscles on T1w and post-gadolinium T1w images were used and a mean SI was calculated for each pair of muscles.

#### 2.3.4 Global muscle T2

Global muscle T2 values were obtained directly from the OsiriX/Horos software. ROIs were drawn for each pair of pelvic limb muscles at the level of the mid-femur on consecutive slices that extended to approximately the length of the femur shaft. Mean global muscle T2 for each pair of pelvic limb muscles (CS, RF, BF, ST, G, AD and VL) were calculated using the slab volume. Sequences were not available for global muscle T2 of lumbar muscles.

#### 2.3.5 Mean left femur length

A 3D multi planar reconstruction of the pelvic limbs from the BALTGRAD sequence was generated for each dog. The length of the left femur was measured from the head of the femur to the medial femoral condyle five times for each animal on the same image and the mean recorded to enable normalization for limb length [Fig. A.1.A.].

#### 2.3.6 L5 length

L5 length was measured on T1w sagittal sequences from the cranial to caudal epiphysis of L5 on the ventral aspect [Fig. A.1.B.]. Five measurements were taken, and the mean recorded to enable normalization for trunk length.

#### 2.3.7 Cranial sartorius muscle circularity

Cranial sartorius muscle circularity was measured bilaterally from a single pelvic limb T1w MRI slice at the level of the mid femur for each WT and DE50-MD dog. Circularity was defined as how close the outline of the CS muscle was to the shape of a circle; ranging from 0.00 to 1.00, where 1 is a perfect circle. A mean circularity value was calculated for each pair of muscles. Single slice images were analyzed in Fiji image processing software (free open source software, imagej.net) [Method C.1].

### 2.4 Musculoskeletal MRI biomarkers

#### 2.4.1 Muscle volume to femur length ratio

Mean muscle volume for each pair of pelvic limb and lumbar muscles normalized to left femur length.

#### 2.4.2 Global muscle T2

Tissue specific quantitative T2w SI values, allowing for direct comparison of global muscle T2 between dogs without normalization.

#### 2.4.3 Ratio of post-gadolinium T1w SI to pre-gadolinium T1w SI

The ratio of post-gadolinium T1w SI to pre-gadolinium T1w SI measures the difference in SI of muscle between pre- and post-gadolinium T1w sequences. It was calculated using mean pre-gadolinium T1w SI and mean post-gadolinium T1w SI for each pair of pelvic limb and lumbar muscles.

$$\begin{array}{ccc} & & \text{Ratio}\;\text{of}\;\text{post-gadolinium}\;\text{to}\;\text{pre-gadolinium}\;\text{T1w}\;\text{SI}\\ & & \;=\frac{\text{Mean}\;\text{post-gadolinium}\;\text{T1w}\;\text{SI}}{\text{Mean}\;\text{pre-gadolinium}\;\text{T1w}\;\text{SI}} \end{array}$$

#### 2.4.4 Lumbar muscle volume to L5 length ratio

The mean muscle volume for each pair of lumbar muscles was normalized to the length of L5.

### 2.5 Statistical analyses

Interclass correlation coefficient (ICC) and Bland-Altman plots were performed to compare absolute normalized muscle volume, global muscle T2, normalized T1w SI and normalized T2w SI between the left and right pelvic limb muscles and left and right lumbar muscles for each group at all ages. Intraclass correlation coefficient (ICC) and Bland Altman plots were performed to assess intra-observer reliability for a repeat set of ROIs drawn on MRI images to original ROIs for absolute muscle volume in a range of pelvic limb and lumbar muscles. A principal component analysis (PCA) was used to summarize variation in musculoskeletal MRI biomarkers among the affected and WT groups and across all ages. The effects of age, genotype and their interaction on the first component (PC1) was then examined statistically using a linear mixed model (LMM) with Fisher's LSD post-hoc comparisons; dog was included as a random effect to account for repeated measures in the LMM analysis. Normality of the residuals was assessed visually using histograms. The same LMM were also used to compare femur length and L5 vertebra lengths between WT and DE50-MD groups, since dog heights/size varied within the colony.

Mean muscle volume to femur length ratio, global muscle T2, ratio of post-gadolinium T1w SI to pre-gadolinium T1w SI, lumbar muscle volume to L5 length ratio and CS muscle circularity were calculated for each pair of muscles and differences examined statistically using the same LMM specifications as the above analyses.

To determine the methodology that may be best utilized in preclinical drug or intervention efficacy studies, we performed statistical sample size calculations (power 0.8, alpha 0.05) on our MRI biomarkers for 25%, 50%, 75% and 100% treatment effect [Table. B.1.].

All statistics were performed using SPSS software (IBM SPSS Statistics 25) or GraphPad Prism 7a (GraphPad Software Inc. 1994–2016) and the results were expressed as means +/-SD unless otherwise stated. Differences and associations were considered statistically significant when *p* < 0.05.

## 3 Results

### 3.1 Qualitative assessment

**Fig. 2:**
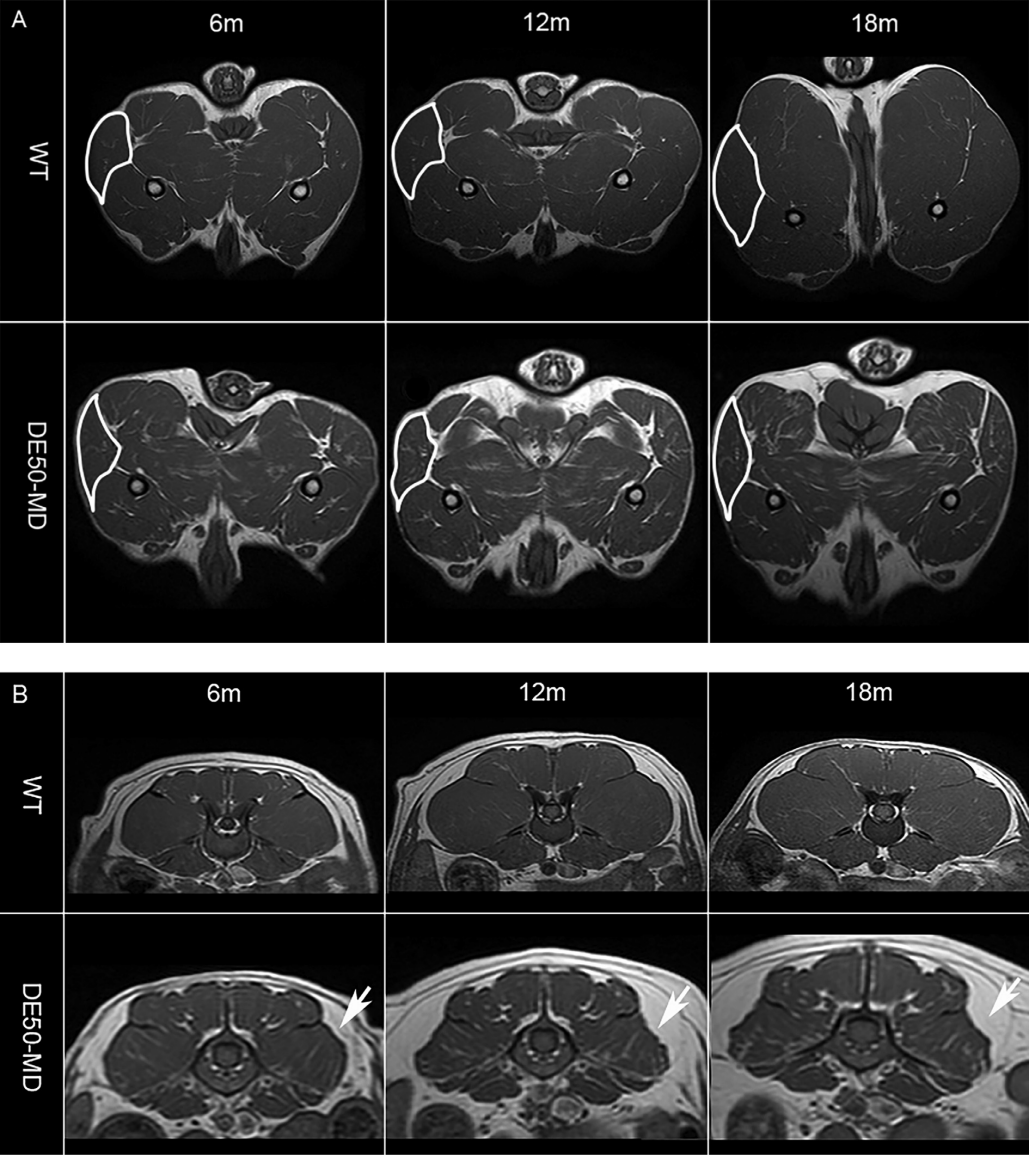
(A) Mid femoral pelvic limb transverse slice T1-weighted MRI images and (B) lumbar spine transverse slice T1-weighted MRI images in a wildtype (WT) and a DE50-MD dogs at multiple time-points; biceps femoris muscles (white outline) were more angular and there was increased fat surrounding lumbar muscles (arrows) in DE50-MD dogs.

**Fig 3:**
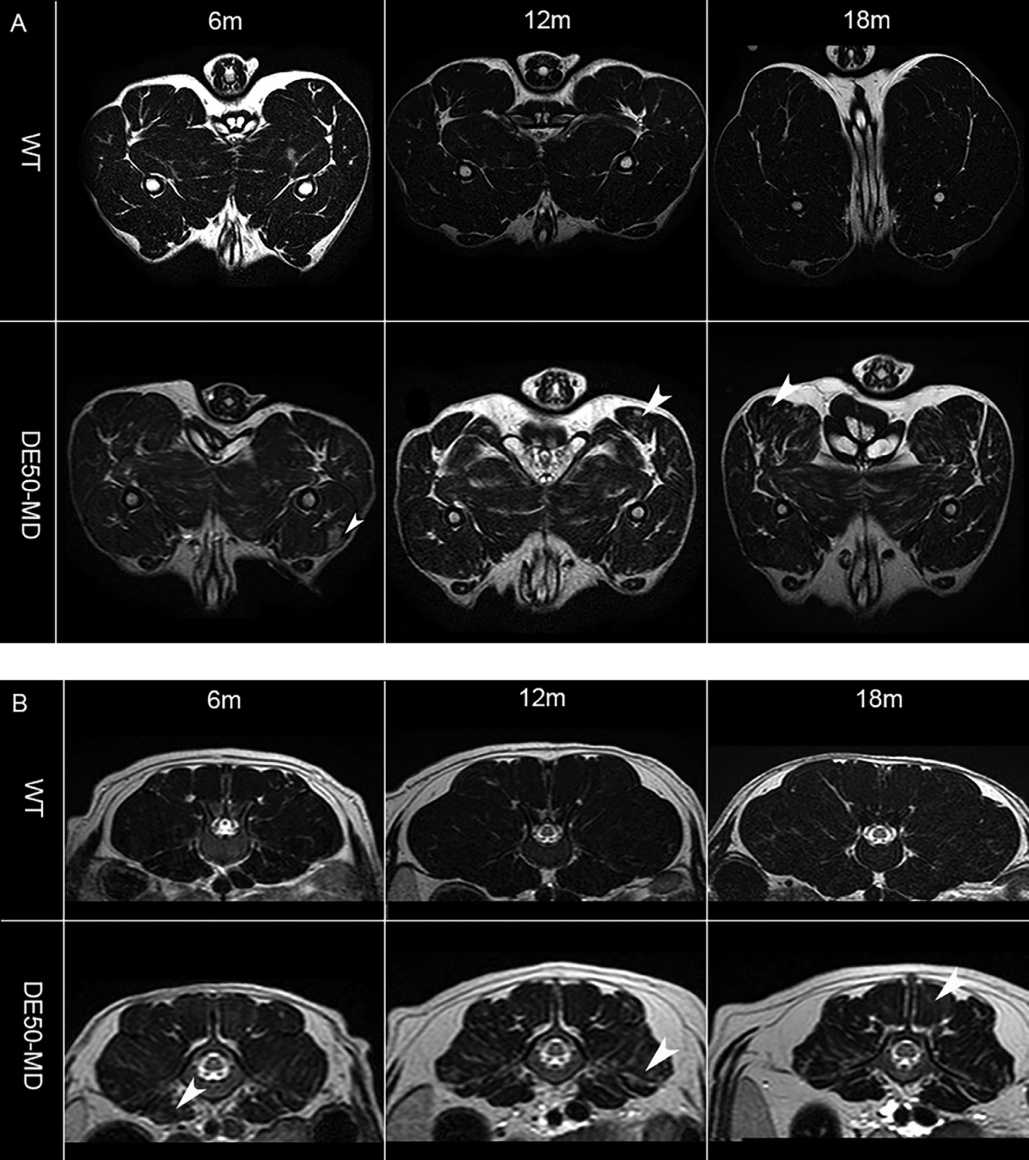
(A) Mid femoral pelvic limb transverse slice T2-weighted MRI and (B) lumbar spine transverse slice T2-weighted MRI images in a wildtype (WT) dog and DE50-MD dog at multiple time-points; arrowheads highlight areas in pelvic limb and lumbar muscles of DE50-MD dogs that are more heterogeneous when compared to the opposite side as well as when compared to WT dogs.

The DE50-MD dog had more angular pelvic limb muscles on each slice, when analyzed visually, compared to WT littermates at the same age, from 6 months old onwards [Fig. 2a.]. As the affected dogs aged, there was an increase in fat surrounding the lumbar muscles [Fig 2b.]. On T2w images, pelvic limb and lumbar muscles in DE50-MD dogs appeared more heterogeneous, especially at later time points, when compared to WT dogs [Fig. 3.a]. Differences were observed between muscles, as well as between limbs or sides of the lumbar spine [Fig. 3.b]. Subjectively, there was no perceivable difference between post- and pre-gadolinium T1w images in DE50-MD dogs [Fig. A.2.A. & A.2.B.]. However, we have further assessed this quantitatively using a ratio of post-gadolinium T1w SI to pre-gadolinium T1w SI.

### 3.2 Quantitative assessment

#### 3.2.1 Comparison between left and right sides

There was very little variation found between left and right pelvic limb and lumbar muscles for absolute muscle volume, global muscle T2, T1w SI and T2w SI in both the DE50-MD group and WT groups [Table B.2.]. Despite the low variation between sides, there was a greater variation between left and right sides for DE50-MD dogs compared with WT dogs across most biomarkers, as seen in Bland Altman plots [Fig. A.3.A. & A.3.B.].

#### 3.2.2 Principal component analysis (PCA)

**Fig 4:**
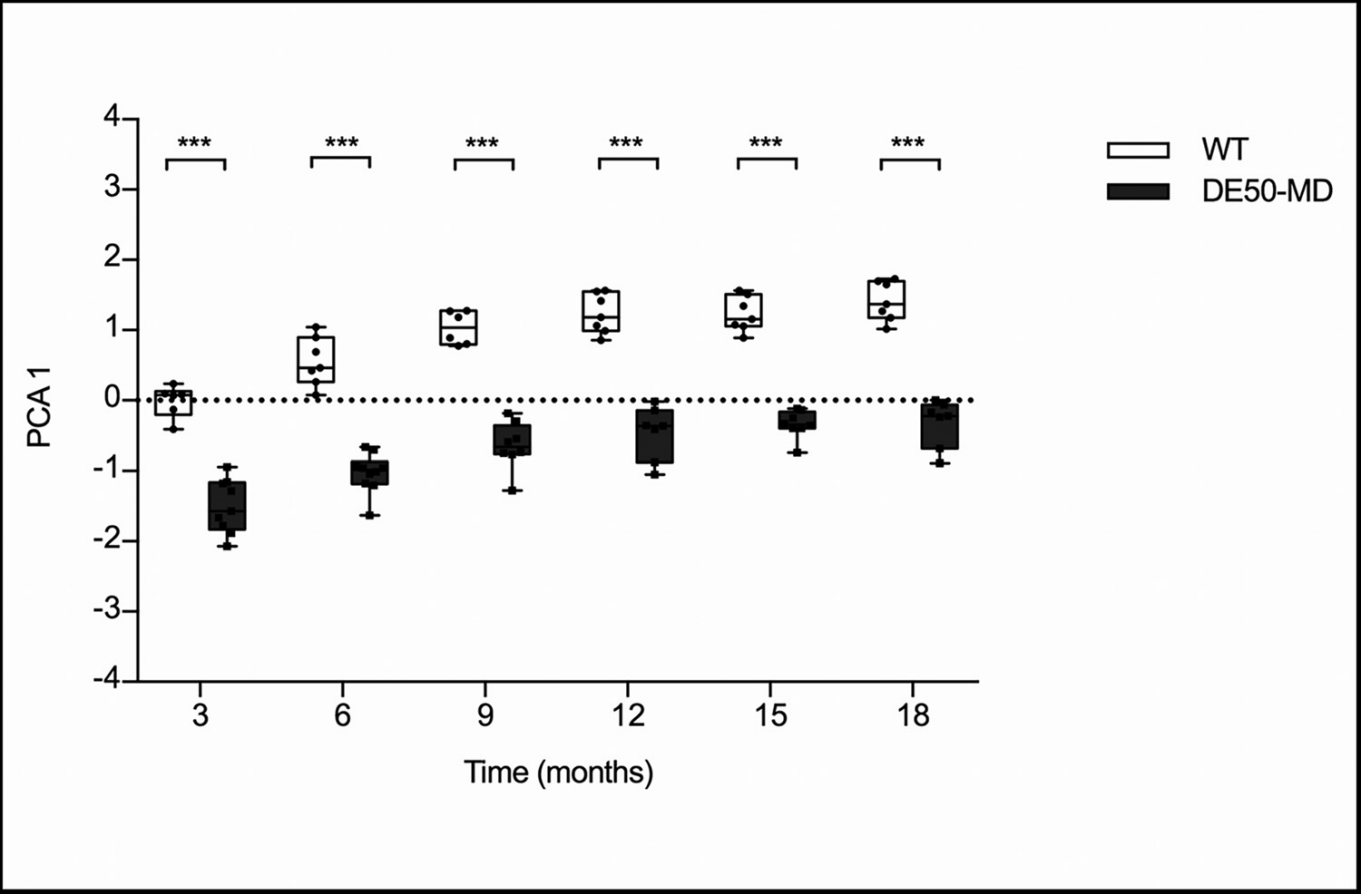
Principal component 1 in wildtype (WT) and DE50-MD dogs from 3-months to 18-months of age (*p*<0.001); points are staggered and not all dogs were included at every time point.

A PCA was run to assess each of the MRI biomarkers for all individual pelvic limb and lumbar muscles. PCA revealed four components that had eigenvalues greater than one and which explained 61.0%, 12.6%, 6.7% and 5.2% of the total variance, respectively. For PC1, there were significant differences between affected DE50-MD dogs (*n* = 10) and WT dogs (*n* = 8, *p*<0.001) at all ages [Fig. 4.]. Muscle volume and global muscle T2 MRI biomarkers contributed most to PC1. There was no effect of interaction between group and time in the remaining components.

#### 3.2.3 Intra-observer reliability in muscle volume ROIs and volumes

There was very little variation found between repeated ROIs drawn and muscle volumes drawn in a randomly selected 5 DE50-MD (ICC = 0.995, *p* < 0.001) and 5 WT (ICC = 0.997, *p* < 0.001) dogs for 5 different pelvic limb and lumbar muscles as seen in Bland Altman plots [Fig. A.4.]

#### 3.2.4 Muscle volume to femur length ratio

**Fig 5:**
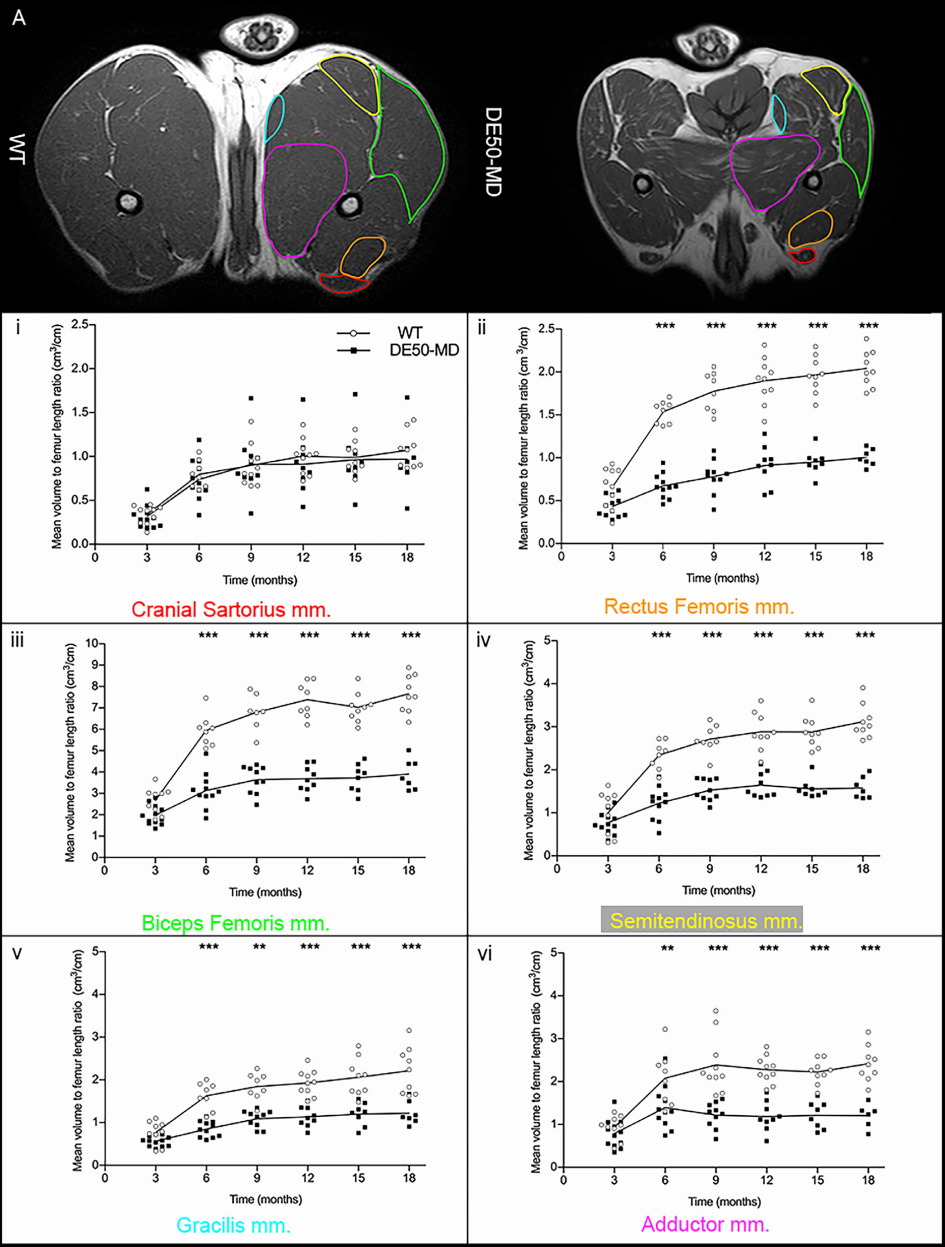

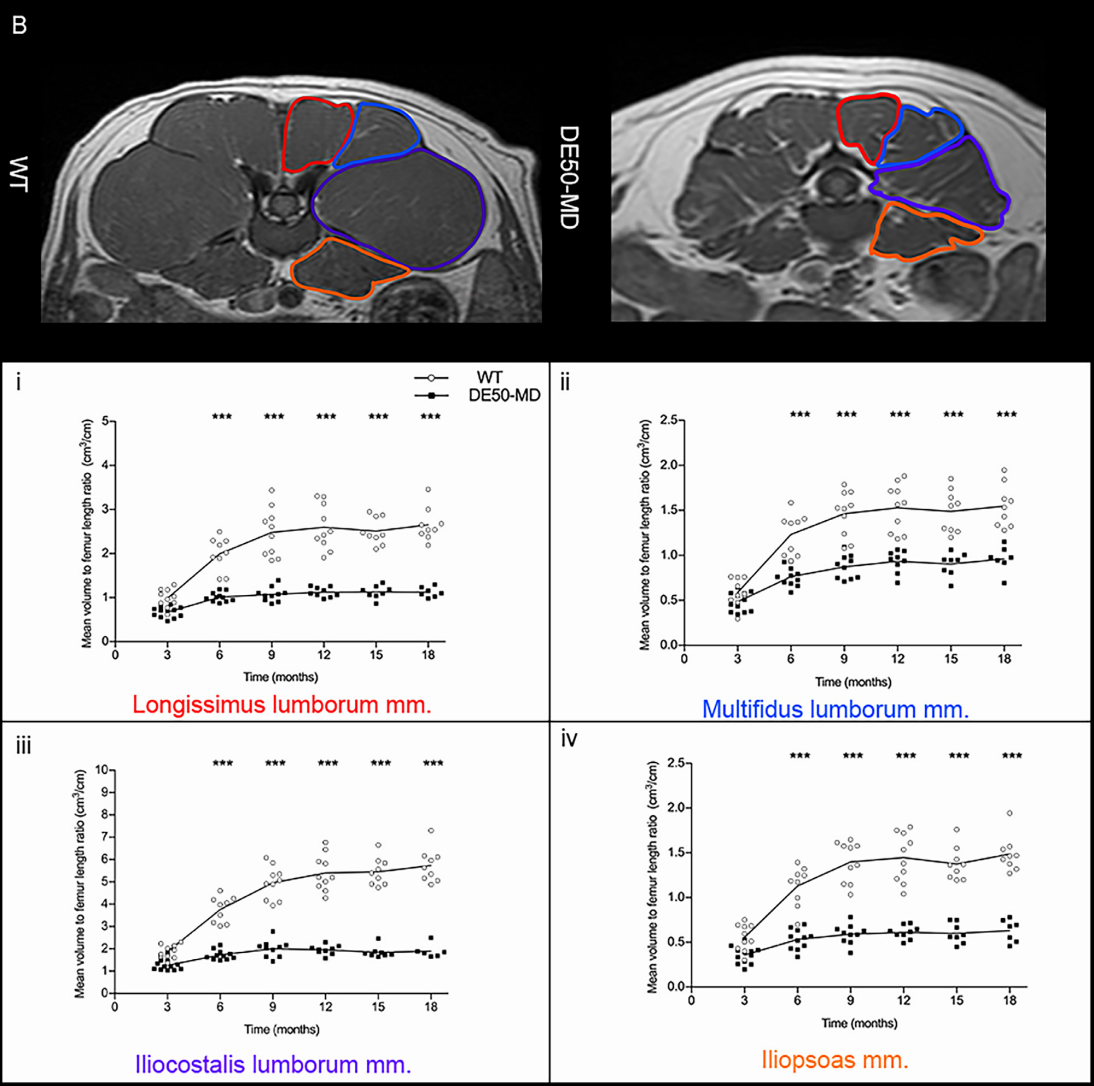
(A) Mid femoral pelvic limb transverse slice T1-weighted MRI images in 12-month-old wildtype (WT) and DE50-MD dogs, outlining pelvic limb muscles; Mean muscle volume normalised to femur length of (i) cranial sartorius muscle (p=0.47), (ii) rectus femoris muscle, (iii) biceps femoris muscle, (iv) semitendinosus muscle, (v) gracilis muscle and (vi) adductor muscle in DE50-MD dogs (n=12) and WT dogs (n=10) every 3 months, from 3 to 18-months of age (**p<0.01, ***p<0.001); points are staggered and not all dogs were included at every time point. (B) Mid L5 transverse slice T1-weighted MRI images in 12-month-old wildtype (WT) and DE50-MD dogs outlining lumbar muscles; Mean muscle volume normalised to femur length of (i) longissimus lumborum muscle, (ii) multifidus lumborum muscle, (iii) iliocostalis muscle and (iv) iliopsoas muscle in DE50-MD dogs (n=12) and WT dogs (n=10) every 3 months, from 3 to 18-months of age (***p<0.001); points are staggered and not all dogs were included at every time point.

There was no significant difference in femur length between WT (mean=10.78, SE= 0.41) and DE50-MD dogs (mean=11.45, SE= 0.34; *p* = 0.22) but there was an effect of age (*p* < 0.001), as expected. Muscle volume for both the pelvic limb and lumbar muscles was normalized to femur length, as previously described in GRMD dogs [26]. There was no significant difference in CS muscle volume to femur length ratio between DE50-MD and WT groups (*p* = 0.47) at all ages [Fig 5a.]. All other pelvic limb and lumbar muscles were significantly smaller in DE50-MD dogs when compared to the WT dogs (*p* < 0.001) from 6 to 18-months old [Fig. 5.A. & 5.B.]. In both groups, the muscle volume to femur length ratio increased as the dogs aged in all muscles, in particular from 6 to 12-months, before plateauing in both groups after 12-months of age.

#### 3.2.5 Global muscle T2

**Fig 6:**
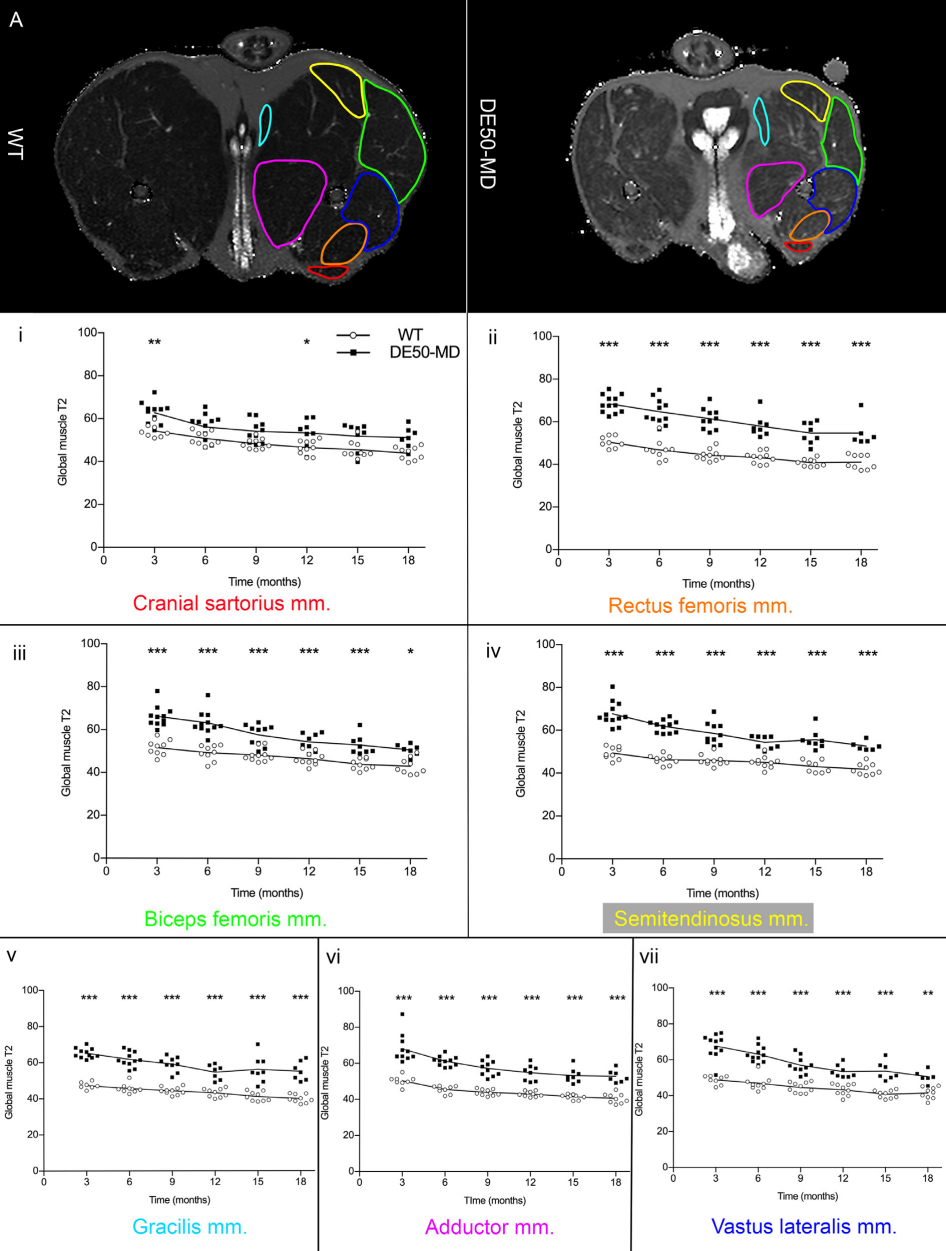
Mid femoral pelvic limb transverse slice multi-slice echo T2-weighted sequence (global muscle T2 map) MRI images in 12-month-old wildtype (WT) and DE50-MD dogs outlining pelvic limb muscles; mean global muscle T2 signal intensity of (i) cranial sartorius muscle, (ii) rectus femoris muscle, (iii) biceps femoris muscle, (iv) semitendinosus muscle, (v) gracilis muscle, (vi) adductor muscle and (vii) vastus lateralis muscle in DE50-MD dogs (n=12) and WT dogs (n=10) every 3 months, from 3 to 18-months of age (*p<0.05, **p<0.01, ***p<0.001); points are staggered at each time point and not all dogs were included at every time point.

In the DE50-MD dogs, global muscle T2 was significantly higher in all pelvic limb muscles at all ages, when compared to WT dogs (*p* < 0.001), with the exception of the CS muscle at 6, 9, 15 and 18-months old (*p* = 0.63) [Fig. 6.]. As dogs aged, the global muscle T2 decreased in both groups.

#### 3.2.6 Ratio of post-gadolinium T1w SI to pre-gadolinium T1w SI

In all pelvic limb and lumbar muscles, apart from in the CS muscle (*p* = 0.22) the post-gadolinium to pre-gadolinium T1w ratio of DE50-MD dogs was significantly higher when compared to WT dogs (*p* < 0.05) at all ages. [Fig. A.5.A. & A.5.B.] However, in all muscles there was no effect of time.

### 3.3 Further quantitative assessment

#### 3.3.1 Lumbar muscle volume to L5 vertebra length ratio

**Fig 7:**
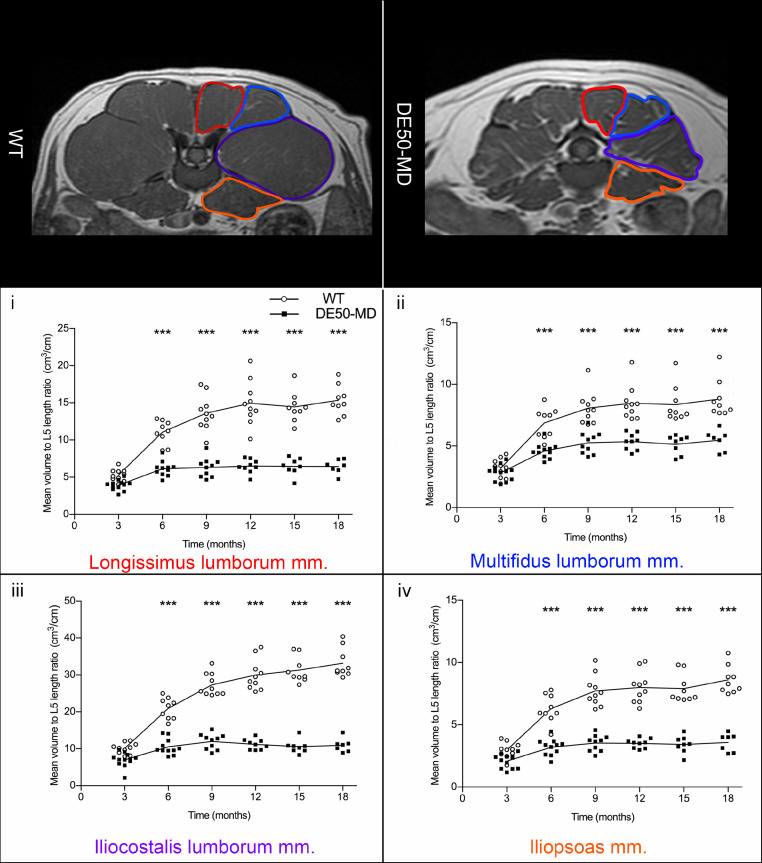
Mid L5 transverse slice T1-weighted MRI images in 12-month-old wildtype (WT) and DE50-MD dogs outlining lumbar muscles; Mean muscle volume normalised to L5 length ratio of the (i) longissimus lumborum muscle, (ii) multifidus lumborum muscle, (iii) iliocostalis lumborum muscle and (iv) iliopsoas muscle in DE50-MD dogs (n=12) and WT dogs (n=10) every 3 months, from 3 to 18-months of age (***p<0.001); points are staggered and not all dogs were included at every time point.

There was no significant difference in L5 length between WT (mean=1.92, SE=0.19) and DE50-MD dogs (mean=1.92, SE=0.19; *p* = 0.96) but there was an effect of age (*p* < 0.001) as expected. All lumbar muscles were significantly smaller in DE50-MD dog when compared to WT dogs (*p* < 0.001) at 6 to 18-months old [Fig. 7.]. In both groups, the muscle volume to L5 length ratio increased as the dogs aged in all muscles, in particular from 6 to 12-months, before plateauing in both groups after 12 months of age.

#### 3.3.2 Cranial sartorius muscle circularity

**Fig 8:**
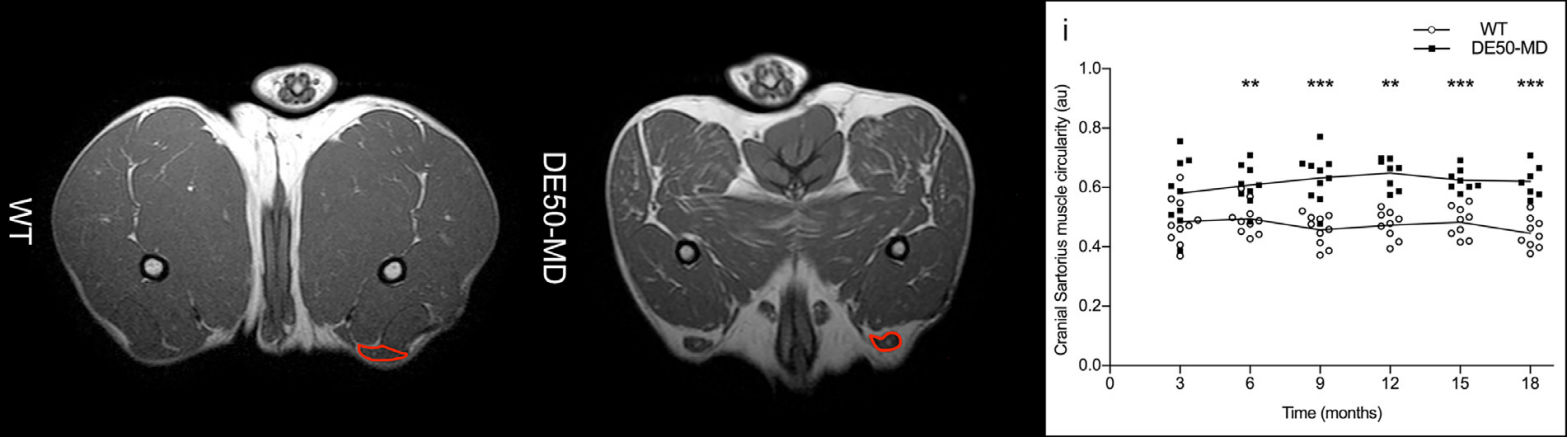
Mid femoral pelvic limb transverse slice T1-weighted MRI images in 12-month-old wildtype (WT) and DE50-MD dogs, outlining the cranial sartorius (red); i) Cranial sartorius muscle circularity in DE50-MD dogs (n=11) and WT dogs (n=10) every 3 months, from 3 to 18-months of age (**p<0.01, ***p<0.001); points are staggered and not all dogs were included at every time point.

The CS muscle was significantly more circular in DE50-MD dogs than in WT dogs (*p* < 0.01) at 6 to 18-months of age [Fig. 8.].

### 3.4 Statistical power calculations

Muscle volume to femur length ratio and global muscle T2 in all pelvic limb muscles, apart from the CS muscle, as well as lumbar muscle volume to L5 length ratio in all lumbar muscles had the smallest sample size for a 50% treatment effect [Table. B.1.].

## 4 Discussion

WT and affected DE50-MD dogs were monitored in this longitudinal natural history MRI study focused on musculoskeletal assessment, from 3 to 18-months of age. There were multiple objectives when undertaking this study with the main focus to determine the most useful non-invasive musculoskeletal MRI biomarkers of disease severity, extent and progression in the DE50-MD dog model. Further, as well as, examining proximal pelvic limb muscles we have examined additional muscle groups (lumbar) that have not previously been examined in canine DMD models.

We have also examined the power/sample size of these analyses for possible future therapeutic trials with the colony. Additionally, we aimed to compare our results in the DE50-MD model with prior published data in longer established DMD dog models and to determine if the DE50-MD dog has a skeletal muscle MRI phenotype that resembles that of DMD boys.

Affected DE50-MD dogs had musculoskeletal MRI biomarker changes that resulted in significant differences when compared to the WT dogs at almost all ages and in nearly all pelvic limb and lumbar muscles. As with the GRMD dog model [21,24], visual analysis of pelvic limb muscles on MRI in affected DE50-MD dogs, revealed more angular shaped muscles, with less rounded edges, when compared to WT dogs.

High ICC correlation coefficients were found between left and right pelvic limb and lumbar muscles in all MRI indices. Bland-Altman plots also determined generally symmetrical changes between the left and right limbs. Therefore, the left and right measurements are not only correlated but also similar in magnitude, which allowed a mean of these MRI biomarker measurements to be compared between groups. This is not unexpected as boys with DMD, as well as animal models, do not show obvious differences between limbs, (even after exercise in the *mdx* mouse) [21,^42^,43]. Femur length was used to normalize pelvic limb muscle volumes, because a previous GRMD study showed that normalizing to femur length was more reliable than normalizing to bodyweight [26]. Using femur length, instead of bodyweight, also compensated for differences in dogs’ heights. Semi-automated segmentation and full-length muscle volume quantification were performed to decrease human error of muscle segmentation [22,25].

As described in GRMD dogs previously [21–26], all pelvic limb muscles, apart from the CS muscle, were smaller in volume in the DE50-MD dog when compared to WT dogs at all ages. The CS muscle in the affected DE50-MD dog appears to be of a similar volume to that of WT dogs, which has also previously been reported in the GRMD dog model. The maintenance of CS volume is achieved by increased circularity: it is possible that this so-called ‘sparing’ [44,45] of the CS muscle in dystrophic dogs, represents the maintenance of muscle volume despite co-existing pathological changes, rather than a muscle that is unaffected. However, histopathological assessment would be required to investigate this possibility. Indeed, in GRMD dogs, the hypertrophied CS muscle showed reduced severity of pathological changes compared to other pelvic limb muscles at 4–6 months of age [44,45]. There was a mild increase in connective tissue within the CS muscle at those early time-points, but as the dogs aged myofibers were more widely separated by fibrosis and fat and the muscle atrophied [44,45]. We speculate that similar changes might occur in this muscle of DE50-MD dogs.

In the DE50-MD dog all lumbar muscles were smaller in volume when compared to WT dogs at all ages. Lumbar muscles have not previously been assessed in any canine model of DMD, perhaps because adding extra MRI sequences could increase general anesthesia duration. Lumbar muscles are not routinely assessed in human DMD patients. Conceivably, this could be due to MRI duration, difficulty positioning when including lumbar sequences or because trunk weakness seems to occur in later disease stages, with the lumbar muscles seemingly more stable in the ambulatory phase [46,47]. Conversely, a more recent study suggests that there is an increased demand on trunk muscles in DMD patients with faster fatigue development and overloading of the muscles [48]. However, because the lumbar muscles have active involvement in locomotion and stability in quadrupeds [49], we examined this additional muscle group in this study. Scan times were not considerably extended [Table 1.], because these relatively small dogs did not need repositioning between sequences. In order to evaluate a size-associated factor in the same musculoskeletal MRI images, we also normalized lumbar muscle volumes to L5 length, rather than only to femur length. L5 length was selected in this study because in dogs, L2 and L5 have less anatomical variation compared to L3, L4 vertebrae (where the diaphragmatic crura attaches) or L1, L7 vertebrae (which are common transitional vertebrae). Therefore, L2 and L5 are commonly used as internal measurement comparisons in veterinary radiology [50,51]. We chose L5 for convenient normalisation of lumbar muscle volume due to its relative position in our dogs to the area of the lumbar spine we were scanning. Lumbar muscle volume normalized to L5 length was a useful MRI biomarker that distinguished DE50-MD dogs from WT dogs. Similarities to the GRMD dog model seen in affected DE50-MD dog pelvic limb muscle volumes were expected, as both canine models follow the same disease progression and see the same pathological changes.

DE50-MD dogs had higher global muscle T2 values in all pelvic limb muscles, apart from the CS muscle, when compared to WT dogs: global muscle T2 increase has previously been reported in boys with DMD and in the GRMD dog model [20,^29^–33], although more recently, global muscle T2 maps have been replaced by Dixon or water T2 maps [20,^34^–40]. Water or fat T2 map SI continues to be the most sensitive marker for observing changes in dystrophic muscle as patients age and when compared to controls [29–32,34,36–40,52]. An increased global muscle T2 is likely to result from inflammation, oedema or fat infiltration of the muscles because T2w SI is lower in muscle than in fat and fluid [53]. In both the DE50-MD and WT group of our dog model there was a gradual decrease in global muscle T2 over time as described in the GRMD dog model [23–26]. We speculate that this decrease over time could be an effect of (1) an increased global muscle T2 value at early age time-points due to oedema and inflammation [53]; (2) a decreased global muscle T2 value with interstitial fibrosis as dogs age [53]; (3) less severely affected dogs reaching later age time points; (4) MSE MRI sequence or (5) muscle fibre type and size variation with age or a combination of these factors. However, we cannot definitively determine the pathological changes without histological comparison.

The contrast agent gadolinium is used more frequently in dogs than in humans, likely because there are concerns about gadolinium toxicity, deposition and retention in humans within the brain and bones, as well as possible deleterious effects of gadolinium on renal function in humans [54,55]. There is limited data in dogs, but a recent small retrospective MRI study indicated there was no visible increased SI in the brain after multiple gadolinium exposures [56]. Most studies of gadolinium-based contrast agents (GBCAs) in animals have been performed in rodents, which confirm similar deposition of gadolinium to humans in the brain, bones and kidneys [57], [58], [59]. In the *mdx* mouse, an intravenous injection of albumin labelled with gadolinium showed contrast enhancement linked to fiber necrosis, identified histologically [60]. However, the clinical significance of gadolinium deposition in humans and animals is yet to be determined.

In our study, post-gadolinium to pre-gadolinium T1w ratio was calculated to highlight our interest in the post-gadolinium SI. The post-gadolinium T1w SI was higher in affected DE50-MD dogs in a few muscles at varying ages when compared to WT dogs, as found in the GRMD dog models [21–27] but with less magnitude. GBCAs can be used to highlight pathological changes or lesions within the musculoskeletal system. A drawback in patients with increased fat deposition, however, is that areas of increased fat have a higher fat SI on pre-gadolinium T1w sequences and can then hide enhancement after gadolinium injection [61]. To rectify this, use of fat suppressed sequences might have been beneficial to enhance the post-gadolinium to pre-gadolinium T1w SI ratio. However, canine models of DMD have decreased fat infiltration when compared to DMD patients due to the early age of boys they represent. Currently, there is little or no use of gadolinium in human DMD studies. Therefore, with no obvious trend over time and limited differences between affected and WT groups in our data, future use of gadolinium contrast agent in the DE50-MD dog model for skeletal muscle assessment is debatable. Especially, when other musculoskeletal MRI biomarkers more effectively demonstrate differences between the two groups [Table B.1.]. If we were to investigate gadolinium contrast agent further in our dog model we would use fat suppressed T1w sequences.

This study was conducted in young dogs which might be considered equivalent in age to DMD boys of approximately 2–5 years old, who typically are still ambulant. Though, histopathological examination is required, this relatively young age might then explain the absence of significant fat infiltration detected by MRI in the DE50-MD dog muscle, something that has also been seen in GRMD dogs [21,^23^,24]. In general, the variation between dogs for musculoskeletal MRI biomarkers, are similar to those reported for the GRMD dog model [Table B.1.] [21,^23^,^24^,26].

Sample size assessment was performed in this study to help determine the most useful musculoskeletal MRI biomarkers to take forward to therapeutic clinical trials in our dog model. Normalized muscle volume and global muscle T2 had the lowest n number for the largest treatment effect size in almost all pelvic limb and lumbar muscles apart from the CS muscle [Table B.1.].

There are limitations of all canine models of DMD when comparing the disease progression and clinical signs to human DMD. For example, oesophageal dysfunction is a canine-specific sign, not commonly seen in boys. Conversely, in DMD boys there is often a loss of ambulation in adolescence due to the dramatic loss of muscle and replacement by fat, which is likely delayed and not as severe in canine models of DMD due to their quadrupedal gait. Contractures can occur in dogs but are less pronounced than in human DMD patients [62]. In addition, welfare and ethical requirements mean that dogs with DMD are euthanized when they reach predefined humane endpoints and prior to the onset of significant musculoskeletal compromise that occurs in human patients.

Musculoskeletal MRI use in patients with DMD is advantageous because it has high sensitivity for identifying early fat replacement in muscles and can be used to monitor disease progression [30,^39^,^40^,[63], [64], [65]. More recently, advanced sequences have been used to determine percentage fat fraction within the muscle; these include Dixon sequences and proton magnetic resonance spectroscopy (MRS). Both these methods aid separation of fat and water signal within muscle and provide outcome measures that are non-invasive for both ambulant and non-ambulant boys and are able to detect significant changes on MRI in patients with excellent reproducibility [34,^35^,^38^,^66^,67]. This is important for disease monitoring because percentage fat fraction has been found to negatively correlate with ambulation in DMD [35]. We could not perform these more advanced MRI sequences on the 1.5T Phillips Intera MRI scanner in our present study. To further examine more advanced musculoskeletal MRI biomarkers in the DE50-MD dog model, such as percentage fat fraction, modified sequences would be needed.

## 5 Conclusions

In conclusion, the most useful and consistent MRI biomarkers found in this study of our unique dog colony were pelvic limb muscle volume normalized to femur length, lumbar muscle volume normalized to L5 length and global muscle T2. These biomarkers provide the best discrimination between DE50-MD and WT groups as they age and will be useful to determine efficacy of therapeutic clinical trials in this dog colony. The DE50-MD dog model from 3 to 18 months of age closely reflects the early MRI phenotype seen in human DMD patients.

## Declarations of Competing Interest

Richard Piercy is a consultant to Exonic Therapeutics; the financial interests have been reviewed and approved by the University in accordance with conflict of interest policies. Studies in his lab have been funded by Pfizer and Exonics Therapeutics. Dominic Wells is or has been a consultant to a wide range of companies with interests in the DMD space including Pfizer, Sarepta, Akashi and Actual Analytics. Studies in his lab have been funded by Proximagen and Shire.

## Appendix Supplementary materials

Image, application 1
