# Supplementary material for: Musculoskeletal magnetic resonance imaging in the DE50-MD dog model of Duchenne muscular dystrophy
Source: Neuromuscul Disord. 2021 Aug;31(8):736–51. doi: 10.1016/j.nmd.2021.05.010 (PMC8449064; doi:10.1016/j.nmd.2021.05.010)
Supplement: Supplementary file 1 [file mmc1.zip › Table B1.docx]

| **MRI Biomarker** | **Muscle** | **Age (months)** | | **DE50-MD mean +/- SD** | **WT mean** | **N per group for 25% treatment effect** | **N per group for 50% treatment effect** | **N per group for 75% treatment effect** | **N per group for 100% treatment effect** |
| --- | --- | --- | --- | --- | --- | --- | --- | --- | --- |
| Muscle volume to femur length ratio (cm^3^/cm) | Cranial Sartorius | 3  6  9  12  15  18 | 0.318+/-0.128  0.739+/-0.237  0.910+/-0.333  0.912+/-0.342  0.964+/-0.358  0.970+/-0.378 | | 0.340  0.774  0.899  1.003  0.989  1.070 | 8368  3983  223905  3524  51447  3585 | 2092  996  55977  881  12862  897 | 930  443  24879  392  5717  399 | 523  249  13995  221  3216  225 |
|  | Rectus Femoris | 3  6  9  12  15  18 | 0.431+/-0.118  0.671+/-0.143  0.781+/-0.195  0.909+/-0.228  0.951+/-0.144  1.003+/-0.099 | | 0.650  1.533  1.651  1.898  1.966  2.040 | 73  9  13  14  6  3 | 19  3  4  4  2  1 | 9  1  2  2  1  1 | 5  1  1  1  1  1 |
|  | Biceps Femoris | 3  6  9  12  15  18 | 1.978+/-0.458  3.139+/-0.805  3.647+/-0.645  3.686+/-0.604  3.733+/-0.644  3.903+/-0.712 | | 2.673  5.946  6.800  7.384  6.915  7.665 | 109  21  11  7  11  9 | 28  6  3  2  3  3 | 13  3  2  1  2  1 | 7  2  1  1  1  1 |
|  | Semi-tendinosus | 3  6  9  12  15  18 | 0.773+/-0.256  1.223+/-0.380  1.525+/-0.250  1.642+/-0.294  1.558+/-0.128  1.576+/-0.251 | | 1.003  2.340  2.717  2.773  1.881  3.121 | 310  30  12  17  7  7 | 78  8  3  5  2  2 | 35  4  2  2  1  1 | 20  2  1  2  1  1 |
|  | Gracilis | 3  6  9  12  15  18 | 0.564+/-0.115  0.849+/-0.187  1.082+/-0.194  1.135+/-0.235  1.198+/-0.268  1.217+/-0.270 | | 0.768  1.628  1.674  1.929  2.060  2.214 | 80  15  27  23  25  19 | 20  4  7  6  7  5 | 9  2  3  3  3  3 | 5  1  2  2  2  2 |
|  | Adductor | 3  6  9  12  15  18 | 0.723+/-0.251  1.330+/-0.321  1.269+/-0.526  1.169+/-0.310  1.227+/-0.332  1.272+/-0.307 | | 0.935  2.083  2.389  2.336  2.156  2.419 | 352  46  56  18  33  18 | 88  12  14  5  9  5 | 40  6  7  2  4  2 | 22  3  4  2  3  2 |
| Muscle volume to L5 length ratio (cm^3^/cm) | Longissimus lumborum | 3  6  9  12  15  18 | 6.962+/-1.953  6.132+/-0.993  6.299+/-1.283  6.482+/-0.924  6.445+/-1.142  6.424+/-0.941 | | 5.284  11.051  13.621  14.946  14.442  15.344 | 340  11  8  3  6  3 | 85  3  2  1  2  1 | 38  2  1  1  1  1 | 22  1  1  1  1  1 |
|  | Multifidus lumborum | 3  6  9  12  15  18 | 2.815+/-0.636  4.596+/-0.614  5.256+/-0.889  5.356+/-0.663  5.129+/-0.762  5.465+/-0.826 | | 3.178  6.874  8.072  8.468  8.361  8.808 | 775  19  26  12  14  16 | 194  5  7  3  4  4 | 87  3  3  2  2  2 | 49  2  2  1  1  1 |
|  | Iliocostalis lumborum | 3  6  9  12  15  18 | 6.962+/-1.953  10.512+/-2.142  11.970+/-2.095  11.176+/-1.400  10.546+/-1.812  10.937+/-1.812 | | 10.105  20.951  27.331  29.975  31.317  33.167 | 97  11  5  2  2  2 | 25  3  2  1  1  1 | 11  2  1  1  1  1 | 7  1  1  1  1  1 |
|  | Iliopsoas | 3  6  9  12  15  18 | 2.077+/-0.555  3.200+-0.666  3.530+/-0.643  3.506+/-0.375  3.422+/-0.691  3.587+/-0.726 | | 2.965  6.291  7.725  7.997  7.910  8.603 | 98  12  6  2  6  6 | 25  3  2  1  2  2 | 11  2  1  1  1  1 | 7  1  1  1  1  1 |
| Global muscle T2 | Cranial Sartorius | 3  6  9  12  15  18 | 62.818+/-5.078  56.140+/-6.081  54.092+/-5.190  53.411+/-6.348  51.796+/-6.421  51.231+/-4.916 | | 54.270  50.798  48.298  46.513  45.560  43.837 | 89  326  202  213  266  111 | 23  82  51  54  67  28 | 10  37  23  24  30  13 | 6  21  13  14  17  7 |
|  | Rectus Femoris | 3  6  9  12  15  18 | 68.515+/-4.343  65.248+/-5.950  61.576+/-4.907  58.164+/-5.178  55.516+/-5.003  54.392+/-6.034 | | 50.468  46.794  44.329  43.168  40.057  41.057 | 15  27  21  30  30  52 | 4  7  6  8  8  13 | 2  3  3  4  4  6 | 1  2  2  2  2  4 |
|  | Biceps Femoris | 3  6  9  12  15  18 | 66.202+/-4.895  63.241+/-5.393  57.663+/-4.939  54.340+/-3.988  52.977+/-4.468  50.743+/-2.602 | | 51.652  49.284  48.181  46.579  43.990  43.154 | 29  38  69  67  63  30 | 8  10  18  17  16  8 | 4  5  8  8  7  4 | 2  3  5  5  4  2 |
|  | Semi-tendinosus | 3  6  9  12  15  18 | 68.331+/-5.451  62.-53+/-2.727  58.961+/-5.035  54.618+/-2.883  55.426+/-4.585  52.779+/-1.960 | | 49.335  46.202  45.951  45.068  42.040  41.820 | 21  8  38  23  30  9 | 6  2  10  6  8  3 | 3  1  5  3  4  1 | 2  1  3  2  2  1 |
|  | Gracilis | 3  6  9  12  15  18 | 65.176+/-2.734  61.777+/-4.143  59.114+/-4.029  54.665+/-4.068  56.239+/-7.347  55.134+/-5.184 | | 47.329  45.642  44.355  43.188  41.151  39.974 | 6  17  19  32  60  30 | 2  5  5  8  15  8 | 1  2  3  4  7  4 | 1  2  2  2  3  2 |
|  | Adductor | 3  6  9  12  15  18 | 68.186+/-7.584  61.028+/-2.746  56.668+/-3.923  54.792+/-4.426  53.167+/-4.040  52.862+/-3.413 | | 50.381  45.540  43.841  43.016  41.450  40.578 | 46  8  24  36  30  20 | 12  2  6  9  8  5 | 6  1  3  4  4  3 | 3  1  2  3  2  2 |
| Ratio of post-gadolinium T1w SI to pre-gadolinium T1w SI | Cranial Sartorius | 3  6  9  12  15  18 | 1.219+/-0.175  1.187+/-0.139  1.198+/-0.161  1.161+/-0.087  1.135+/-0.117  1.131+/-0.105 | | 1.106  1.176  1.145  1.131  1.124  1.121 | 596  39400  2366  1990  25451  28221 | 149  9850  592  498  6363  7056 | 67  4378  263  222  2828  3136 | 38  2463  148  125  1591  1764 |
|  | Rectus Femoris | 3  6  9  12  15  18 | 1.343+/-0.158  1.364+/-0.178  1.386+/-0.185  1.372+/-0.131  1.275+/-0.086  1.268+/-0.134 | | 1.127  1.159  1.132  1.102  1.128  1.104 | 135  189  134  60  87  169 | 34  48  34  15  22  43 | 15  21  15  7  10  19 | 9  12  9  4  6  11 |
|  | Biceps Femoris | 3  6  9  12  15  18 | 1.322+/-0.137  1.253+/-0.131  1.253+/-0.170  1.240+/-0.119  1.160+/-0.098  1.169+/-0.079 | | 1.131  1.172  1.163  1.112  1.175  1.128 | 130  660  900  215  10627  954 | 33  165  225  54  2657  239 | 15  74  100  24  1181  106 | 9  42  57  14  665  60 |
|  | Semi-tendinosus | 3  6  9  12  15  18 | 1.340+/-0.170  1.252+/-0.122  1.273+/-0.138  1.258+/-0.108  1.190+/-0.103  1.188+/-0.103 | | 1.123  1.153  1.143  1.112  1.110  1.110 | 155  378  280  139  424  428 | 39  95  70  35  106  107 | 18  42  32  16  48  48 | 10  24  18  9  27  27 |
|  | Gracilis | 3  6  9  12  15  18 | 1.281+/-0.137  1.205+/-0.142  1.211+/-0.135  1.197+/-0.094  1.161+/-0.123  1.163+/-0.139 | | 1.139  1.123  1.149  1.107  1.128  1.107 | 233  745  1184  278  3470  1526 | 59  187  296  70  868  382 | 26  83  132  31  386  170 | 15  47  74  18  217  96 |
|  | Adductor | 3  6  9  12  15  18 | 1.341+/-0.131  1.295+/-0.155  1.313+/-0.217  1.273+/-0.106  1.215+/-0.109  1.210+/-0.105 | | 1.109  1.146  1.140  1.123  1.128  1.117 | 80  271  395  126  393  320 | 20  68  99  32  99  80 | 9  31  44  14  44  36 | 5  17  25  8  25  20 |
|  | Longissimus lumborum | 3  6  9  12  15  18 | 1.320+/-0.156  1.261+/-0.089  1.316+/-0.113  1.240+/-0.110  1.210+/--0.081  1.277+/-0.295 | | 1.176  1.168  1.168  1.108  1.044  1.074 | 293  230  147  175  61  528 | 74  58  37  44  16  132 | 33  26  17  20  77  59 | 19  15  10  11  4  33 |
|  | Multifidus lumborum | 3  6  9  12  15  18 | 1.323+/-0.161  1.264+/-0.094  1.291+/-0.117  1.170+/-0.055  1.150+/-0.048  1.230+/-0.272 | | 1.177  1.155  1.1333  1.105  1.044  1.073 | 304  186  138  183  52  757 | 76  47  35  46  13  190 | 34  21  16  21  6  85 | 19  12  9  12  4  48 |
|  | Iliocostalis lumborum | 3  6  9  12  15  18 | 1.317+/-0.143  1.255+/-0.072  1.319+/-0.110  1.240+/-0.116  1.192+/-0.052  1.279+/-0.288 | | 1.178  1.153  1.157  1.098  1.030  1.083 | 265  124  117  169  26  542 | 67  31  30  43  7  136 | 30  14  13  19  3  61 | 17  8  8  11  2  34 |
|  | Iliopsoas | 3  6  9  12  15  18 | 1.348+/-0.166  1.252+/-0.187  1.295+/-0.095  1.236+/-0.110  1.192+/-0.073  1.266+/-0.206 | | 1.210  1.206  1.188  1.134  1.070  1.131 | 360  4034  199  292  91  578 | 90  1009  50  73  23  145 | 40  449  23  33  11  65 | 23  253  13  19  6  37 |

**Supplementary Table B.1.** Sample size results of all MRI biomarkers for all pelvic limb and lumbar muscles (power 0.8, alpha 0.05). The most useful and consistent MRI biomarkers are muscle volumes and global muscle T2**.**
