# Supplementary material for: Musculoskeletal magnetic resonance imaging in the DE50-MD dog model of Duchenne muscular dystrophy
Source: Neuromuscul Disord. 2021 Aug;31(8):736–51. doi: 10.1016/j.nmd.2021.05.010 (PMC8449064; doi:10.1016/j.nmd.2021.05.010)
Supplement: Supplementary file 1 [file mmc1.zip › Table B2.docx]

| **Group** | **Muscle** | **ICC for muscle volume (cm^3^)** | **ICC for global muscle T2** | **ICC for T2w SI** | **ICC for T1w SI** |
| --- | --- | --- | --- | --- | --- |
| WT | Cranial sartorius | 0.989 | 0.786 | 0.912 | 0.913 |
|  | Rectus femoris | 0.993 | 0.826 | 0.922 | 0.932 |
|  | Biceps femoris | 0.994 | 0.750 | 0.940 | 0.619 |
|  | Semitendinosus | 0.989 | 0.791 | 0.949 | 0.774 |
|  | Gracilis | 0.981 | 0.902 | 0.971 | 0.987 |
|  | Adductor | 0.950 | 0.936 | 0.950 | 0.987 |
|  | Vastus lateralis | - | 0.561 | - | - |
|  | Longissimus lumborum | 0.980 | - | - | 0.884 |
|  | Multifidus lumborum | 0.996 | - | - | 0.979 |
|  | Iliocostalis lumborum | 0.992 | - | - | 0.819 |
|  | Iliopsoas | 0.991 | - | - | 0.857 |
| DE50-MD | Cranial sartorius | 0.994 | 0.883 | 0.964 | 0.963 |
|  | Rectus femoris | 0.985 | 0.815 | 0.952 | 0.926 |
|  | Biceps femoris | 0.991 | 0.925 | 0.968 | 0.915 |
|  | Semitendinosus | 0.985 | 0.775 | 0.900 | 0.930 |
|  | Gracilis | 0.982 | 0.877 | 0.967 | 0.987 |
|  | Adductor | 0.950 | 0.944 | 0.983 | 0.992 |
|  | Vastus lateralis | - | 0.874 | - | - |
|  | Longissimus lumborum | 0.982 | - | - | 0.881 |
|  | Multifidus lumborum | 0.985 | - | - | 0.980 |
|  | Iliocostalis lumborum | 0.985 | - | - | 0.832 |
|  | Iliopsoas | 0.966 | - | - | 0.947 |

**Supplementary Table B.2.** Interclass correlation coefficients (ICC) of normalised muscle volume, global muscle T2, T2w SI and T1w SI between the left and right pelvic limb and lumbar muscles for each group at all ages calculated using linear mixed model. There was very little variation found between the left and right pelvic limb and lumbar muscles; all values were significant to p<0.001.
