# Supplementary material for: Musculoskeletal magnetic resonance imaging in the DE50-MD dog model of Duchenne muscular dystrophy
Source: Neuromuscul Disord. 2021 Aug;31(8):736–51. doi: 10.1016/j.nmd.2021.05.010 (PMC8449064; doi:10.1016/j.nmd.2021.05.010)
Supplement: Supplementary file 1 [file mmc1.zip › Figure and Table Legends_V1.docx]

**Appendix A: Supplementary Figure legends**

**Supplementary Figure A.1.** **(A)** Mid femoral coronal slice dorsal T2-weighted thin slice gradient echo (BALTGRAD) MRI image of a 12-month-old wildtype dog showing the method of femur length measurement (green line) from head of femur to medial femoral condyle; **(B)** Lumbar spine sagittal slice T1-weighted image of a 12-month-old wildtype dog showing the method of L5 vertebra length measurement (green line) from the cranial to caudal edge of L5 vertebra on the ventral aspect.

**Supplementary Figure A.2. (A)** Mid femoral pelvic limb transverse slice T1-weighted pre-gadolinium and T1w post-gadolinium MRI images in wildtype (WT) and DE50-MD dogs at 12-months-old; **(B)** Lumbar spine transverse slice T1-weighted pre-gadolinium and post-gadolinium MRI images in WT and DE50-MD dogs at 12-months-old. This has been further assessed quantitatively using a ratio of pre- to post-gadolinium T2w signal intensity (SI).

**Supplementary Figure A.3. (A)** Bland Altman plots of **(i)** pelvic limb muscle volume in wildtype (WT) dogs and **(ii)** pelvic limb muscle volume in DE50-MD dogs; **(iii)** pelvic limb global muscle T2 in WT dogs and **(iv)** pelvic limb global muscle T2 in DE50-MD dogs; **(v)** pelvic limb T2w signal intensity in WT dogs and **(vi)** pelvic limb T2-weighted signal intensity in DE50-MD dogs; **(vii)** pelvic limb T1-weighted signal intensity in WT dogs and **(viii)** pelvic limb T1-weighted signal intensity in DE50-MD dogs.

**Supplementary Figure A.3. (B)** Bland Altman plots of **(i)** lumbar muscle volume in wildtype (WT) dogs and **(ii)** lumbar muscle volume in DE50-MD dogs; **(iii)** lumbar muscle T1-weighted signal intensity in WT dogs and **(iv)** lumbar muscle T1-weighted signal intensity in DE50-MD dogs.

**Supplementary Figure A.4.** Bland Altman plots of **(i)** pelvic limb and lumbar muscle volumes in WT dogs and **(ii)** pelvic limb and lumbar muscle volumes in DE50-MD dogs.

**Supplementary Figure A.5. (A)** Mid femoral pelvic limb transverse slice post-gadolinium T1-weighted MRI images in 12-month-old wildtype (WT) and DE50-MD dogs outlining pelvic limb muscles; Mean ratio of post-gadolinium T1-weighted signal intensity to pre-gadolinium T1-weighted signal intensity of **(i)** cranial sartorius muscle, **(ii)** rectus femoris muscle, (**iii)** bicep femoris muscle, **(iv)** semitendinosus muscle, **(v)** gracilis muscle and **(vi)** adductor muscle in DE50-MD dogs (n=11) and WT dogs (n=10) every 3 months, from 3 to 18-months of age (*<0.05, **<0.01, ***p<0.001); points are staggered and not all dogs were included at every time point.

**Supplementary Figure A.5. (B)** Mid L5 vertebra transverse slice post-gadolinium T1-weighted MRI images in 12-month-old wildtype (WT) and DE50-MD dogs outlining lumbar muscles; Mean ratio of post-gadolinium T1-weighted signal intensity to pre-gadolinium T1-weighted signal intensity of **(i)** longissimus lumborum muscle, **(ii)** multifidus lumborum muscle, (**iii)** iliocostalis muscle and **(iv)** iliopsoas muscle in DE50-MD dogs (n=11) and WT dogs (n=10) every 3 months, from 3 to 18-months of age (*<0.05, **<0.01, ***p<0.001); points are staggered and not all dogs were included at every time point.

**Appendix B: Supplementary Table Legends**

**Supplementary Table B.1.** Sample size results of all MRI biomarkers for all pelvic limb and lumbar muscles (power 0.8, alpha 0.05). The most useful and consistent MRI biomarkers are muscle volumes and global muscle T2**.**

**Supplementary Table B.2.** Interclass correlation coefficients (ICC) of normalised muscle volume, global muscle T2, T2w SI and T1w SI between the left and right pelvic limb and lumbar muscles for each group at all ages calculated using linear mixed model. There was very little variation found between the left and right pelvic limb and lumbar muscles; all values were significant to p<0.001.

**Appendix C: Supplementary Methods**

**Supplementary Method C.1.** Cranial sartorius (CS) muscle circularity

1. Single slice pelvic limb T1w MRI images at the level of the mid femur were uploaded to Fiji image processing software (free open source software, imagej.net).
2. The image black and white threshold was adjusted to highlight the outline of the CS muscle bilaterally. It was adjusted manually as needed and mask created.
3. Particles were analysed setting particle size to 20-infinity, circularity to 0.00-1.00 and outlines were selected.
4. A circularity value was recorded from the correct outlines on the drawing for each CS muscle and a mean calculated for each pair of muscles.
