# Supplementary figures and images for: Musculoskeletal magnetic resonance imaging in the DE50-MD dog model of Duchenne muscular dystrophy
Source: Neuromuscul Disord. 2021 Aug;31(8):736–51. doi: 10.1016/j.nmd.2021.05.010 (PMC8449064; doi:10.1016/j.nmd.2021.05.010)

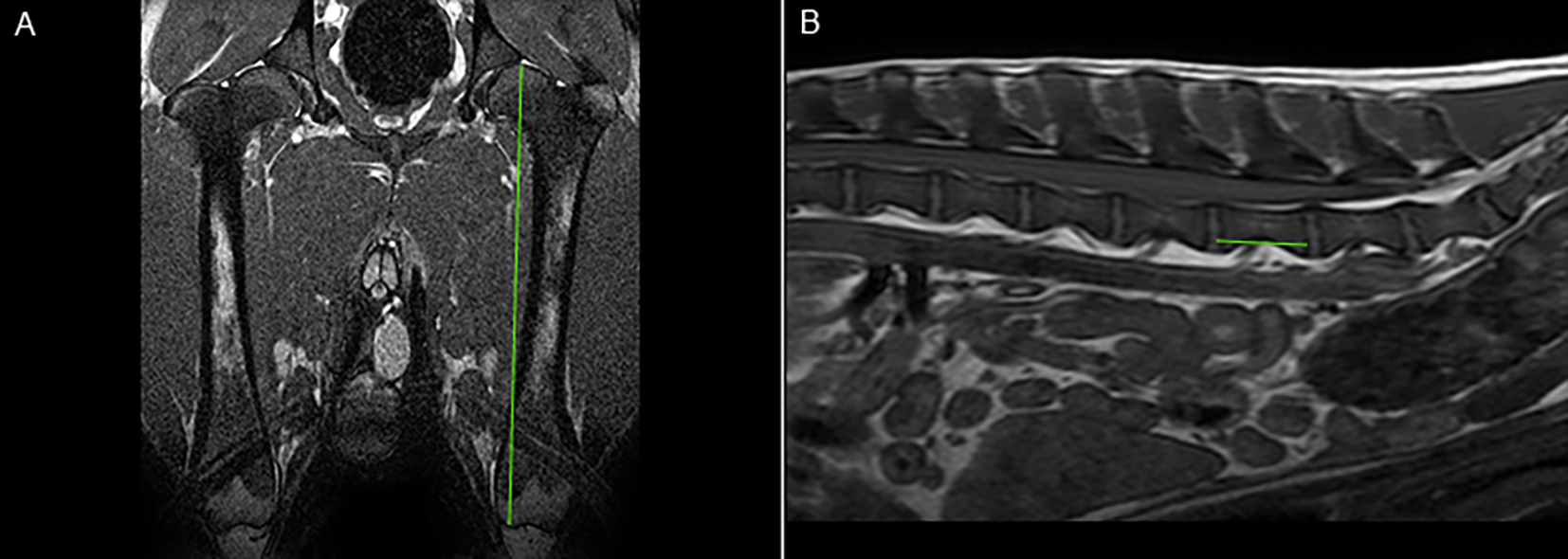

Supplement: Supplementary file 1 [file mmc1.zip › Fig A1.tif]

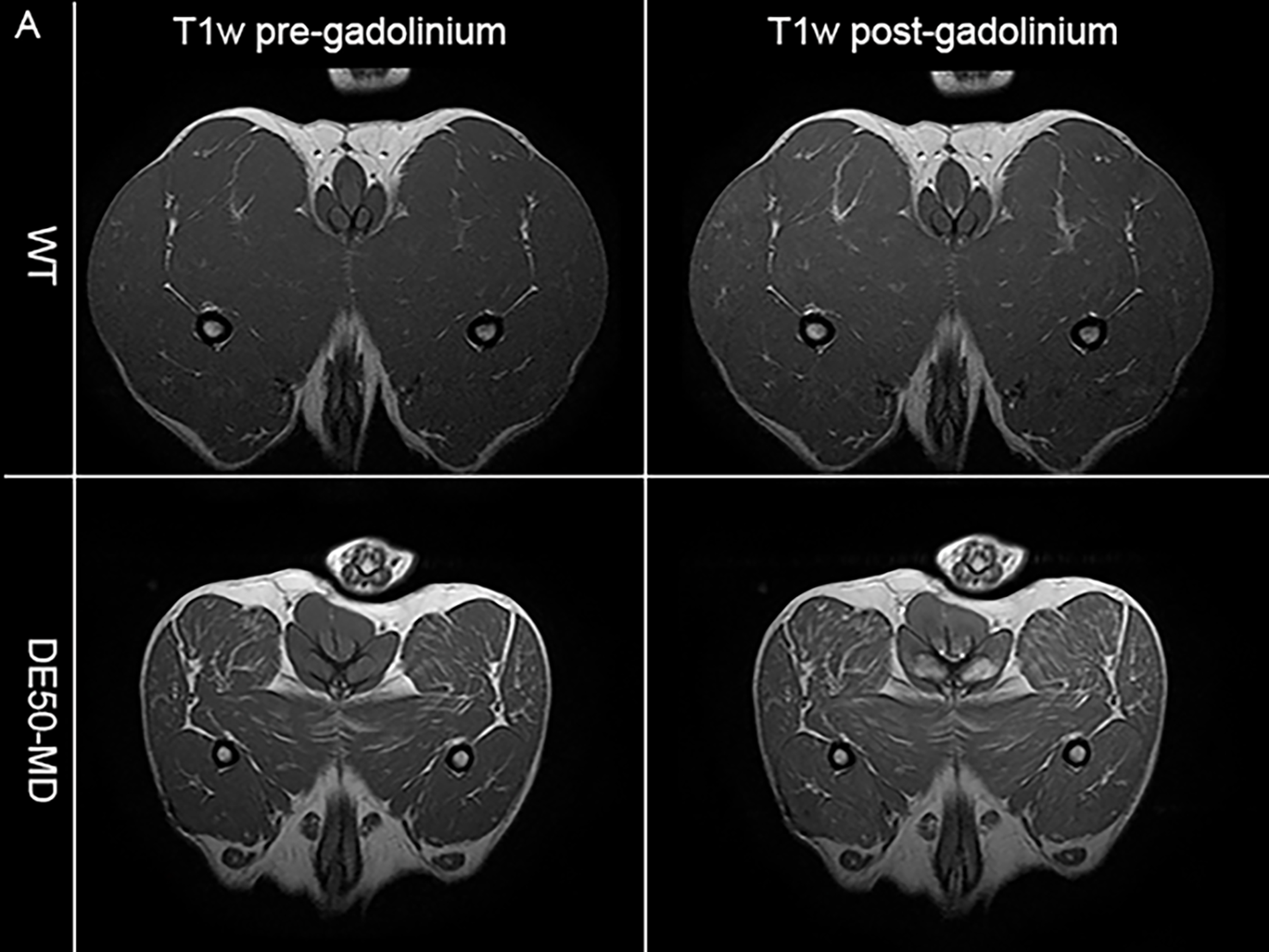

Supplement: Supplementary file 1 [file mmc1.zip › Fig A2A.tif]

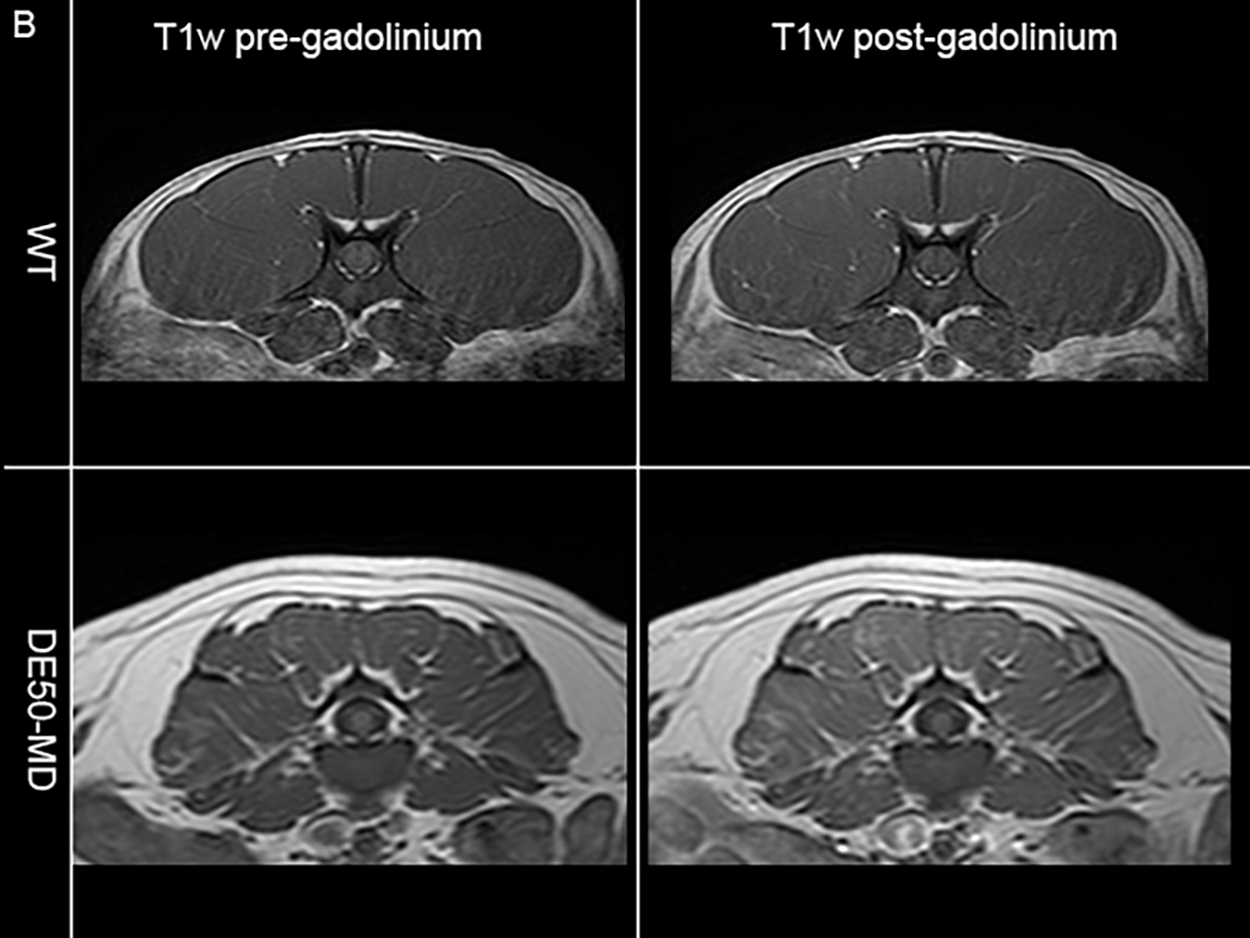

Supplement: Supplementary file 1 [file mmc1.zip › Fig A2B.tif]

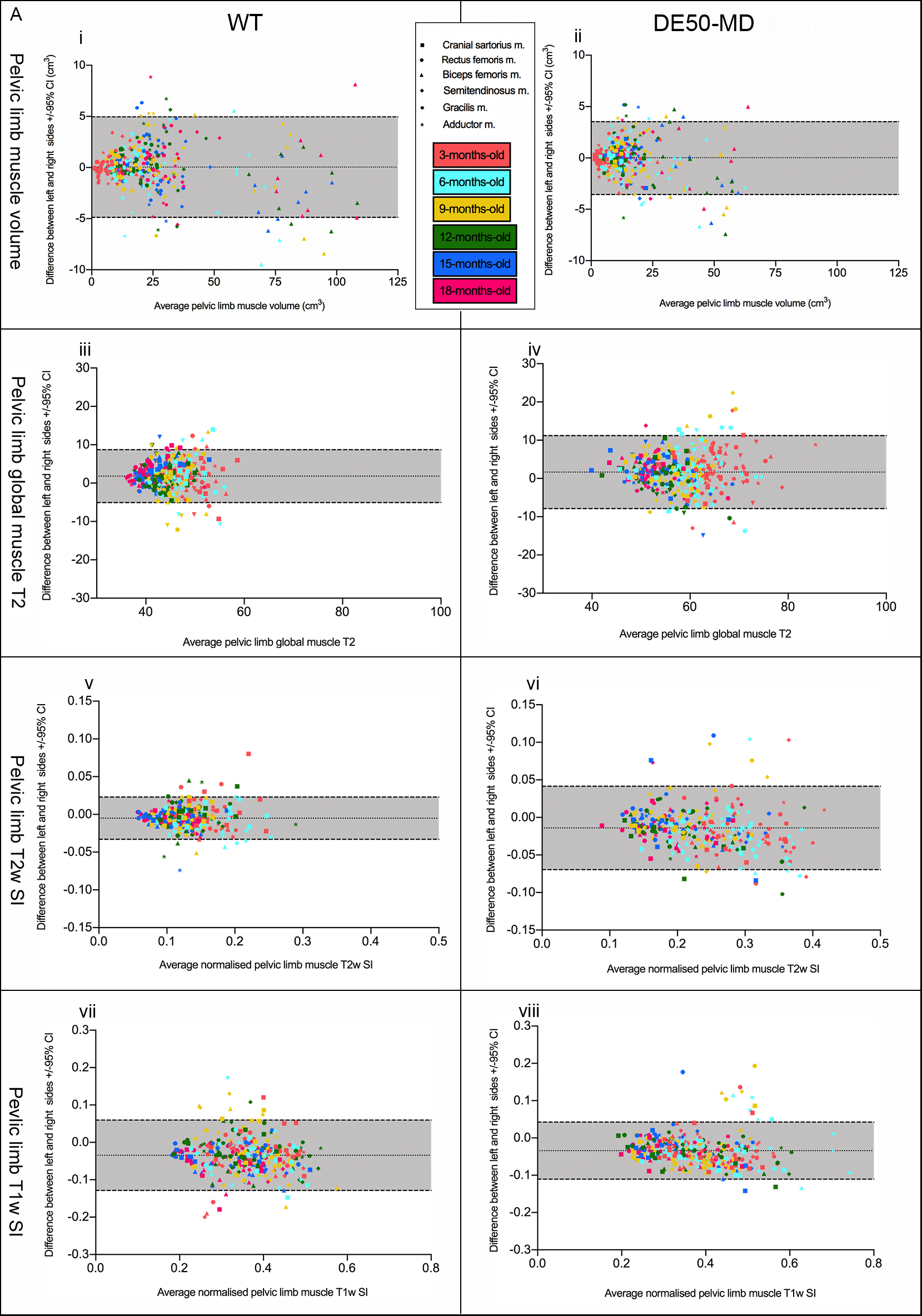

Supplement: Supplementary file 1 [file mmc1.zip › Fig A3A.tif]

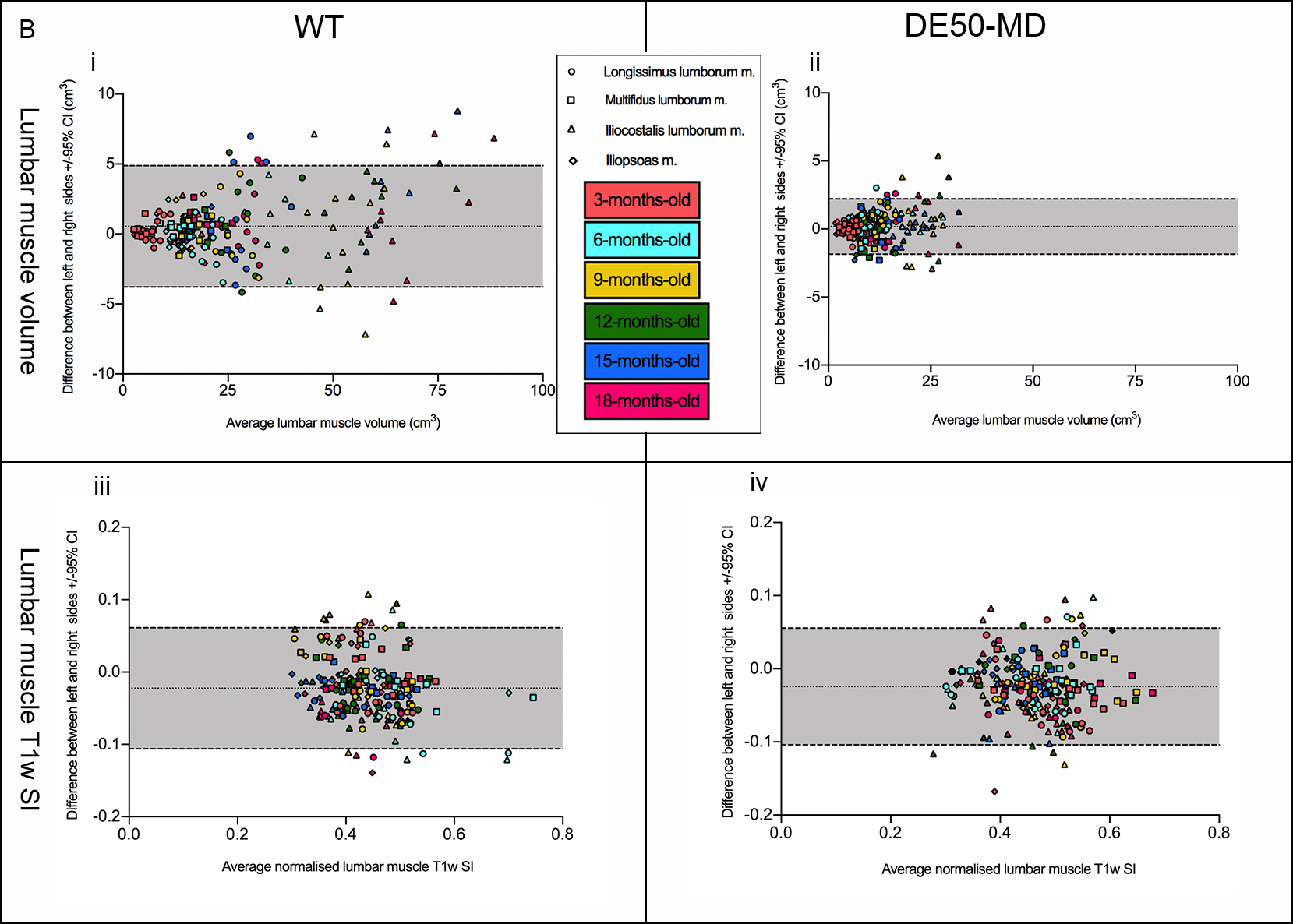

Supplement: Supplementary file 1 [file mmc1.zip › Fig A3B.tif]

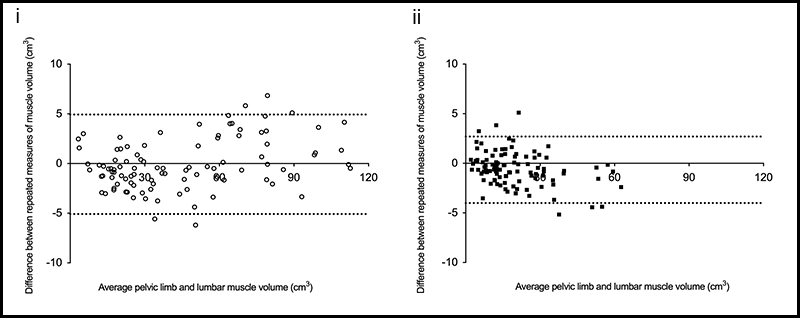

Supplement: Supplementary file 1 [file mmc1.zip › Fig A4.tif]

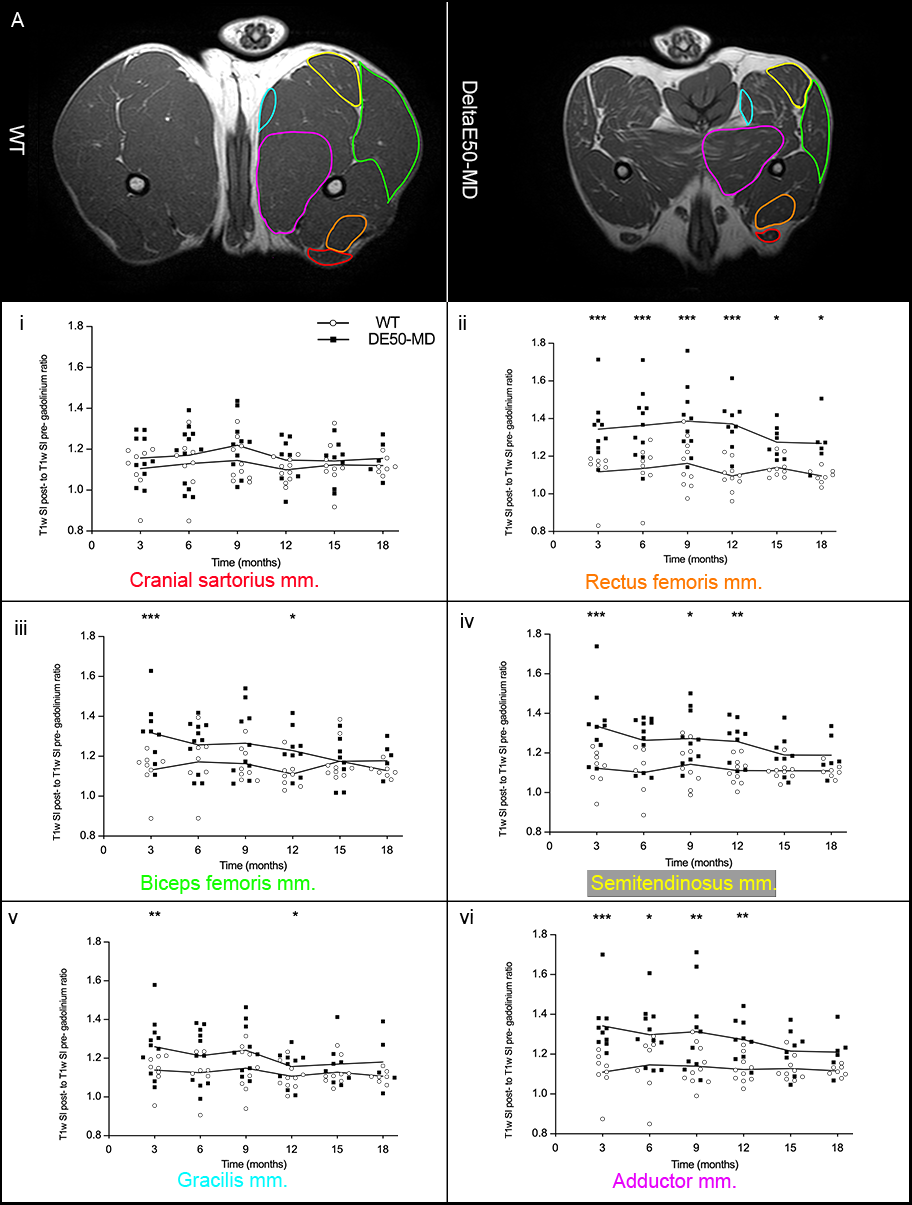

Supplement: Supplementary file 1 [file mmc1.zip › Fig A5A.tif]

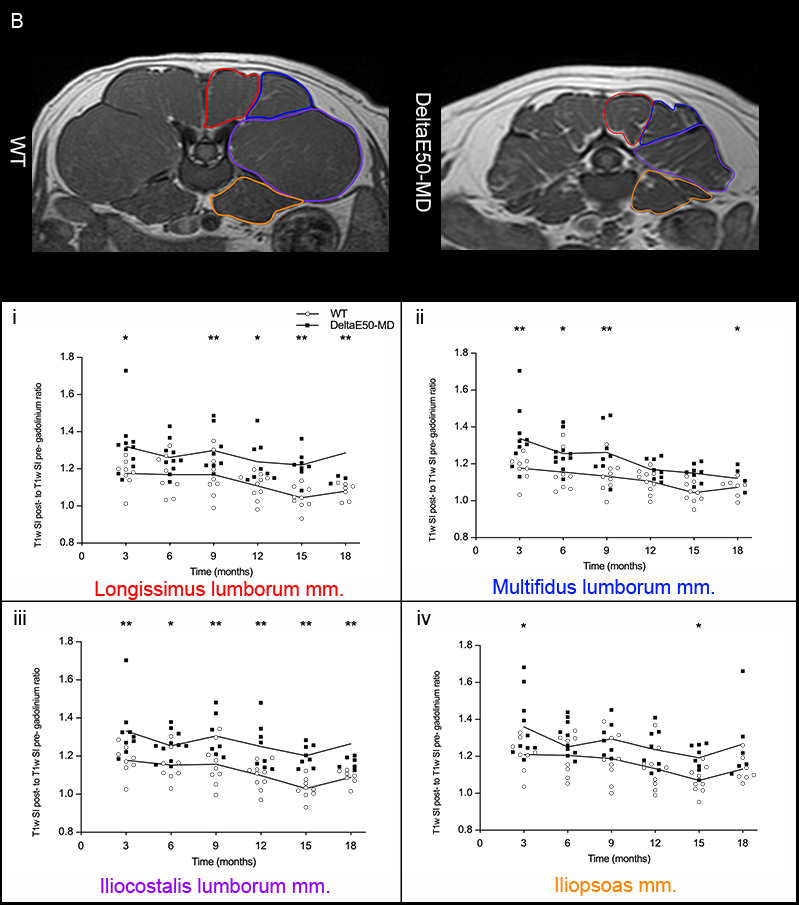

Supplement: Supplementary file 1 [file mmc1.zip › Fig A5B.tif]
